# ECM stiffness inhibits ferroptosis to promote HCC progression via the FOXA1-CDON-HMGB1/p53 pathway

**DOI:** 10.7150/ijbs.129038

**Published:** 2026-10-09

**Authors:** Qianben Song, Xilin Ye, Xin Mao, Lumiao Zhang, Mengjia Jing, Yu Fu, Wei Yan

**Affiliations:** 1 Department of Gastroenterology, Tongji Hospital, Tongji Medical College, Huazhong University of Science and Technology, Wuhan City, Hubei, 430022, PR China.; 2 Department of Gastroenterology, Union Hospital, Tongji Medical College, Huazhong University of Science and Technology, Wuhan City, Hubei, 430022, PR China.

**Keywords:** extracellular matrix, stiffness, hepatocellular carcinoma, CDON, ferroptosis

## Abstract

The progression of hepatocellular carcinoma (HCC) is closely associated with the physical microenvironment, particularly the stiffness of the extracellular matrix (ECM). However, the molecular mechanisms by which the ECM influences HCC progression remain unclear. We demonstrate that a stiff ECM promotes HCC progression and identify the key regulatory molecule CDON using a polyacrylamide hydrogel model, transcriptome sequencing, and other assays. A gradual increase in ECM stiffness upregulates CDON expression, and elevated CDON levels are associated with poor prognosis. CDON is highly expressed in HCC cells and tissues, where it enhances invasion, metastasis, and proliferation. ECM stiffness regulates HCC progression through CDON. In the cytoplasm, CDON binds to HMGB1, reducing HMGB1 levels in both the cytoplasm and nucleus. This reduction leads to the release of p53 from the nucleus into the cytoplasm and its degradation through the ubiquitination-proteasome pathway, weakening p53's transcriptional repression of SLC7A11 and thereby inhibiting ferroptosis, which promotes sorafenib chemotherapy resistance in HCC cells. Additionally, stiff ECM promotes the nuclear translocation of YAP1 via integrins. Once in the nucleus, YAP1 binds to the transcription factor FOXA1 to regulate CDON transcription. This study demonstrates that stiff ECM serves as an indicator for differentiating HCC progression and chemotherapy resistance. Targeting the "CDON-ferroptosis" axis may offer a novel combined treatment strategy.

## Introduction

Hepatocellular carcinoma (HCC) is one of the leading causes of cancer-related deaths worldwide, and its treatment remains challenging, especially for advanced patients and those with chemotherapy resistance 1. In recent years, research has expanded beyond cancer cells themselves to focus on the critical role of the tumor microenvironment. Among the various components of the tumor microenvironment, pathological remodeling of the extracellular matrix (ECM) is especially prominent, with ECM stiffness significantly increasing during the progression of liver fibrosis and liver cancer 2. This increased ECM stiffness is not merely a passive scaffold; it actively promotes tumor cell proliferation, invasion, and immune evasion through mechanotransduction signaling 3. Studies have shown that softening the ECM can reverse cancer cell malignancy and enhance drug sensitivity 4. Therefore, clarifying how ECM stiffness regulates the malignant phenotype and therapeutic resistance of liver cancer cells is expected to facilitate the development of new therapies.

Identifying the key molecules that mediate mechanical signal transduction of the ECM is a core issue in the field of mechanical biology of liver cancer. This study focuses on CDON, a receptor crucial for embryonic development, as it affects limb growth, oligodendrocyte differentiation, myelin formation, and endothelial cell integrity 5-7. Studies have shown that CDON is highly expressed in prostate cancer. The deficiency of CDON inhibits the apoptosis and invasion of prostate cancer cells 8. CDON promotes proliferation and inhibits apoptosis in non-small cell lung cancer. Additionally, it is associated with an increased risk of ovarian cancer development 9, 10. CDON expression is elevated in pancreatic cancer; the absence of CDON and its co-receptors (Gas1 and Boc) in cancer-associated fibroblasts leads to impaired tumor promotion and angiogenesis 11. Furthermore, CDON deficiency has been shown to reduce activation and/or cause mislocalization of integrin β1 12. Integrins, heterodimers composed of α and β subunits, are essential for cells to sense changes in ECM stiffness. ECM stiffness drives chemotherapy resistance and angiogenesis through the integrin β1-Yes-associated protein 1 (YAP1) or integrin-Rac1-YAP1 pathways 13, 14. It suggests that CDON might serve as a new type of mechanical sensitivity factor. These findings indicate that CDON plays a pro-oncogenic role in multiple cancer types. However, its role in liver cancer and its alterations within the HCC microenvironment under specific stiffness conditions remain unclear.

Among various modes of cell death, ferroptosis—a type of iron-dependent regulated cell death triggered by the lethal accumulation of lipid peroxides on the cell membrane—has attracted significant attention due to its potential to overcome chemotherapy resistance 15. The process of ferroptosis involves iron accumulation, the generation of free radicals, dysfunction of the antioxidant system, and lipid peroxidation. The core regulatory component of ferroptosis, the SLC7A11/SLC3A2 complex, forms the cystine/glutamate antiporter (system xc-), which transports cystine to promote the biosynthesis of glutathione (GSH) and antioxidant activity 16. The commonly used chemotherapy drug sorafenib, a ferroptosis inducer, can inhibit the activity of the xc- system, reducing cysteine uptake by HCC cells and the synthesis of GSH 17, 18. Ferritin heavy chain 1 (FTH1) enhances iron storage in HCC cells and confers resistance to ferroptosis 19. ACSL4 dictates PUFA-phospholipid composition and ferroptosis sensitivity 20. As the main antioxidant transcription factor, NRF2 activates the SLC7A11-GPX4 axis, FSP1, and FTH1, while inhibiting ACSL4 21. Fe^2+^ acts as a cofactor for lipoxygenase (LOX), generating active iron oxide and amplifying membrane lipid peroxidation 22.

Interestingly, the stiff ECM can inhibit ferroptosis in HCC cells by promoting the expression of SLC3A2 and GPX4 in these cells 23. ECM stiffening in HCC induces the expression of PD-L2, thereby impairing the ferroptosis of HCC cells mediated by SLC7A11 24. These findings demonstrate that the stiffness of the ECM might be an important factor contributing to increased chemotherapy resistance in cancer. However, relatively few studies have examined the influence of ECM stiffness on the regulation of ferroptosis and sorafenib resistance in liver cancer cells. This article explores this topic.

Overall, this study systematically elucidates the signaling axis from ECM stiffness to intracellular ferroptosis resistance. We first identified CDON as the central molecule in this process: The integrins on HCC cells sense changes in the stiffness of the ECM, promoting the dephosphorylation of the transcriptional co-activator YAP1 and its translocation to the nucleus. YAP1, together with the transcription factor FOXA1, targets CDON, thereby promoting the upregulation of CDON transcription. CDON not only drives the proliferation, invasion, and metastasis of HCC cells. More importantly, it enhances the expression of SLC7A11 to inhibit ferroptosis by regulating the spatial distribution of HMGB1-p53 complexes within the nucleus and modulating the interaction between HMGB1 and p53. This mechanism collectively promotes malignant progression and sorafenib resistance in HCC. The findings of this study deepen our understanding of tumor mechanobiology and propose targeting the "CDON-ferroptosis" axis as a novel combined therapeutic strategy, offering new perspectives and potential targets for overcoming stroma-driven resistance in HCC.

## Results

### Stiff ECM promotes the progression of liver cancer

We know that most clinical cases of liver cancer result from the gradual progression of liver fibrosis and cirrhosis to liver cancer 25. As shown in Figure S1A, we randomly divided C57 mice into two groups: the control group received intraperitoneal injections of olive oil, and the liver fibrosis model group received intraperitoneal injections of carbon tetrachloride, both for 8 weeks. Then, we performed an orthotopic xenograft HCC model using Hep1-6 cells and conducted subsequent observations for three weeks. As shown in Figure S1B, the results of HE and Masson staining indicated that the liver fibrosis model was successfully established after 8 weeks of carbon tetrachloride injection. As shown in Figure S1C, the orthotopic xenograft tumors in the liver cirrhosis background mice progressed more rapidly. These findings suggest that liver tissue stiffening promotes liver cancer progression. However, since mice in the liver fibrosis group exhibited not only significantly increased liver stiffness but also alterations in the hepatic inflammatory environment, we subsequently used polyacrylamide hydrogels to simulate changes in ECM stiffness.

The flowchart for the preparation of polyacrylamide hydrogel is shown in Figure 1A. We cultured the HCC cell lines MHCC97H and Huh7 on collagen-coated polyacrylamide hydrogels with elastic moduli of 0.48 ± 0.16 kPa (“soft”) and 10.61 kPa (“stiff”). After 72 hours, the cells were digested for colony formation assays, Cell Counting Kit-8 (CCK-8) assays, wound healing assays, and Transwell assays. The results showed that the stiff ECM not only promoted the proliferation of HCC cells *in vitro* but also facilitated their migration and invasion (Figure 1B-E). Studies have demonstrated that diluting the hydrogel stock solution in different proportions allows it to be used with liver cancer cells, injected into mice, and tested in a variety of environments with varying stiffness levels 26. As shown in Figure 1F, we mixed the stably transfected firefly luciferase MHCC97H cells with hydrogels of different stiffnesses and established an orthotopic mouse xenograft model. Starting 2 weeks after the surgery, we conducted live imaging observations of the tumor progression in the mice every week. In the fifth week, we immediately sacrificed the mice after taking live imaging, then quickly isolated the mice's livers, immediately performed live imaging on the livers, and took a macroscopic view of the mice's livers. The results showed that tumor progression in mice's livers was faster under the stiff ECM (Figure 1G-H). The liver tumors were larger, and the *in vivo* imaging signals were higher (Figure 1I). To rule out the influence of liver tumor signals on the tumor signals of lung metastatic nodules, when we sacrificed the mice, we also quickly isolated the lungs of the mice and immediately performed *in vivo* imaging of the lungs. The results showed that the stiff ECM promoted lung metastasis of liver cancer (Figure 1J-K). To further clarify, we performed HE staining on the lungs and observed that mice in the stiff ECM treatment group had pulmonary metastatic nodules (Figure 1L). The above results indicate that stiff ECM can promote the progression of liver cancer both *in vivo* and *in vitro*.

### CDON is overexpressed in stiff ECM and promotes the progression of HCC *in vitro*

In order to investigate the specific molecular mechanism by which the stiff ECM promotes the progression of HCC, we cultured MHCC97H cells on different stiffness levels of polyacrylamide hydrogels (0.48 ± 0.16 kPa, 2.01±0.75kPa, 10.61 kPa) for 72 hours, collected the cell extracts for RNA, and sent them for transcriptome sequencing. The results showed that 29 genes exhibited increased molecular expression as the ECM stiffness increased (Figure 2A-B). Then, we conducted a prognostic analysis using the Kaplan-Meier Plotter, and the results indicated that 5 genes encoding proteins (CDON, RBP5, SYNE2, DGKH, PLEKHG1) were associated with the prognosis of liver cancer patients (*P*<0.05) (Figure S2A-B). Higher expression of CDON in patients with liver cancer was significantly correlated with poorer prognosis, whereas the remaining four genes showed a protective prognostic pattern. This opposite prognostic direction led us to prioritize CDON for further functional characterization, as it most plausibly represents a stiffness-induced pro-tumorigenic factor. At the same time, we drew a heatmap of these five genes (Figure 2C). Thus, we speculate that CDON may be a key molecule for the promotion of liver cancer progression by the stiff ECM.

We cultured MHCC97H and Huh7 cells on different stiffness hydrogels (0.48 ± 0.16 kPa, 2.01±0.75kPa, 10.61 kPa) for 72 hours for qRT-PCR and Western blot assays for verification. The results showed that the expression of CDON gradually increased in the stiff ECM (Figure 2D-E). To figure out whether CDON expression follows a linear or threshold-based response at higher pathological stiffness ranges, we have now performed additional Western blot experiments examining CDON protein levels across a comprehensive stiffness gradient: tissue culture plastic (TCP, GPa range, representing a supra-physiological stiff reference), 0.48 ± 0.16 kPa, 2.01±0.75 kPa, 10.61 kPa, 19.66 ±1.19 kPa, and 40.40 ± 2.39 kPa. The results demonstrate a clear threshold-based, rather than linear, response pattern: CDON expression is minimally detectable on 0.5 kPa substrates, consistent with our original observations. A robust and significant upregulation of CDON protein is observed at 10 kPa, which closely approximates the clinical cirrhotic threshold. Importantly, CDON expression does not continue to increase proportionally with further stiffening. On 20 kPa and 40 kPa substrates, CDON protein levels remain elevated but do not significantly differ from those observed at 10 kPa, indicating a plateau phase. This pattern suggests that CDON expression is governed by a stiffness-sensing mechanism that is "switched on" once a permissive mechanical threshold is exceeded, rather than being linearly modulated by incremental stiffness increases (Figure S2C). This further confirmed that the stiff ECM could promote the expression of CDON. Secondly, we analyzed the TCGA data through the UALCAN website (https://ualcan.path.uab.edu/), and the results showed that the expression of CDON was elevated in liver cancer (Figure S3A). We extracted RNA and proteins from normal liver cells (THLE2 and THLE3) and liver cancer cells (Huh7, SNU-398, MHCC97H, PLC/PRF/5) and verified them through qRT-PCR and Western blot assays. The findings indicated an increased expression of CDON in liver cancer cells (Figure S3B-C). We evaluated CDON expression in clinical samples of liver cancer and adjacent non-tumor tissues using Western blot analysis. The results revealed that CDON was significantly upregulated in liver cancer tissues compared with adjacent tissues. (Figure S3D). Immunohistochemical analysis further demonstrated that CDON exhibited elevated expression levels in liver cancer (Figure S3E-F). The result of combining the IHC score with clinical prognosis analysis showed that patients exhibiting elevated levels of CDON expression were associated with a poorer prognosis (Figure S3G). In univariate Cox regression analysis, high CDON expression (HR = 3.241, 95% CI: 1.827-6.132, *P* < 0.0001), poor differentiation (HR = 1.908, 95% CI: 1.114-3.263, *P* = 0.019) and age(>52.5(median)) (HR = 2.292, 95% CI: 1.359-3.920,* P* = 0.002) were significantly associated with worse overall survival, while AFP (>20 ng/mL) showed no prognostic significance (Figure S3H and Table S1). However, in the multivariate Cox model, AFP (>20 ng/mL) (HR = 1.769, 95% CI: 1.033-3.026, *P* = 0.038) unexpectedly emerged as a significant independent predictor of poor survival (Figure S3I). This phenomenon suggests a potential suppression effect, wherein the prognostic impact of AFP was initially masked by its positive correlation with other factors, such as CDON expression, in the univariate analysis. We used GEPIA and Kaplan-Meier plotter to draw Disease Free Survival and KM curves, and the results showed that patients exhibiting elevated levels of CDON expression were associated with a poorer prognosis (Figure S3J). In conclusion, CDON is highly expressed in liver cancer and is an independent prognostic risk factor along with low differentiation degree, advanced age, and high AFP levels.

Based on the expression of CDON in various cell lines mentioned earlier. MHCC97H cells, which exhibited the second-highest CDON expression and robust invasive/tumorigenic capacity, were selected for knockdown experiments, while Huh7 cells with low basal CDON expression were used for overexpression assays (Figure S3B-C). Verification by Western blot assays and qRT-PCR showed that the cell lines MHCC97H-shCDON and Huh7-LVCDON were successfully constructed (Figure 2F-H). We conducted a colony formation assay and CCK-8 using the constructed MHCC97H-shCDON and Huh7-LVCDON. The results indicated that altering CDON expression affected the proliferative capacity of HCC cells (Figure 2I-J). We also conducted wound healing assays, Transwell migration and invasion assays, and the results indicated that alterations in CDON expression affected the invasion and metastasis ability of HCC cells (Figure 2K-L). In conclusion, CDON promotes the progression of liver cancer *in vitro*.

### Stiff ECM promotes the progression of HCC by regulating the expression of CDON

To verify the role of CDON in stiff ECM, the MHCC97H-shCDON and Huh7-LVCDON HCC cell lines were respectively cultured in polyacrylamide hydrogels of different stiffness for 72 hours, followed by digestion and colony formation assays, CCK-8, wound healing assays, and Transwell assays. The results showed that stiff ECM promoted the proliferation, invasion, and metastasis of HCC cells. Knockdown of CDON could weaken the promoting effect of a stiff ECM on the proliferation, invasion, and metastasis of liver cancer cells, while overexpression of CDON could weaken the inhibitory effect of soft ECM on the proliferation, invasion, and metastasis of HCC cells (Figure 3A-D).

To verify the role of CDON in stiff ECM *in vivo*, we mixed MHCC97H-shCDON cells stabilized with firefly luciferase with hydrogels of different stiffness to conduct the mouse orthotopic xenograft model. The results indicated that the stiff ECM promoted the progression of liver cancer, and knocking down the expression of CDON in the stiff ECM could delay the growth of liver cancer (Figure 3E-F). We isolated the mouse lungs for living image, counted the incidence of liver cancer lung metastasis in the various groups, and fixed and wax-coated the isolated lungs for HE staining. The results showed that the stiff ECM promoted liver cancer lung metastasis, and knocking down the expression of CDON in the stiff ECM could delay liver cancer lung metastasis (Figure 3G-H). Immunohistochemical results showed that the proliferation marker protein ki67 and the angiogenesis marker protein CD31 were expressed at higher levels in stiff ECM, and knocking down the expression of CDON in the stiff ECM led to a decrease in the expression of ki67 and CD31(Figure 3I). We also mixed Huh7-LVCDON cells expressing firefly luciferase with hydrogels of different stiffness to establish an orthotopic mouse xenograft model. The results indicated that the softer ECM inhibited liver cancer growth, and overexpressing CDON in the softer ECM could exacerbate liver cancer growth (Figure 3J-K). We also isolated the mouse lungs for a living image and counted the incidence of liver cancer lung metastasis in the various groups. The isolated lungs were then fixed, embedded in paraffin, sectioned, and stained with HE. The findings indicated that the incidence of liver cancer lung metastasis was lower in the softer ECM, and overexpression of CDON could increase the incidence of liver cancer lung metastasis in the softer ECM (Figure 3L-M). In conclusion, the stiff ECM promotes liver cancer progression through CDON.

### CDON inhibits ferroptosis in HCC cells

Studies have shown that a stiff ECM can inhibit ferroptosis in HCC cells 24. Inhibition of ferroptosis in HCC cells can promote the progression of HCC 27-29. Our research indicates that a stiff ECM can promote the progression of HCC by regulating the expression of CDON. Then, can stiff ECM affect ferroptosis in HCC cells by regulating CDON? We used kits to detect ferroptosis-related indicators (GSH, ROS, MDA, ferrous ions) in MHCC97H and Huh7 HCC cells treated with different ECM stiffness levels (0.48 ± 0.16 kPa and 10.61 kPa). The findings indicated that a stiff ECM reduced the consumption of GSH and the levels of ROS, the lipid peroxidation degradation product MDA, and ferrous ions (Figure 4A). We conducted tests on ferroptosis-related indicators (GSH, ROS, MDA, ferrous ions) in the MHCC97H-shCDON and Huh7-LVCDON HCC cell lines. The results indicated that overexpression of CDON reduced the consumption of GSH and decreased the levels of ROS, MDA, and ferrous ions. Knockdown of CDON led to a large consumption of GSH and increased the levels of ROS, MDA, and ferrous ions (Figure 4B).

We treated MHCC97H-shCDON and Huh7-LVCDON HCC cell lines with the ferroptosis-specific inhibitor Ferrostatin-1 (Fer-1) and the ferroptosis inducer Erastin. The CCK-8 results indicated that knocking down CDON inhibited the proliferation of HCC cells, whereas Fer-1 promoted their proliferation. Under ferroptosis inhibition conditions, CDON expression decreased, which inhibited the proliferation of HCC cells; conversely, overexpression of CDON promoted cell proliferation, and Erastin inhibited it. Under ferroptosis induction, increased CDON expression enhanced liver cancer cell proliferation (Figure 4C). The C11-BODIPY staining results showed that knocking down CDON increased the lipid peroxidation levels in liver cancer cells, while Fer-1 reduced the lipid peroxidation. CDON knockdown increases lipid peroxidation under Fer-1 treatment (Figure 4D-E). Overexpression of CDON alleviated lipid peroxidation, whereas Erastin induced lipid peroxidation in liver cancer cells. CDON overexpression markedly attenuated Erastin-induced lipid peroxidation, an effect that was reversed by the ferroptosis inhibitor Fer-1 (Figure 4F-G).

However, the proliferation inhibition and elevated lipid peroxidation caused by CDON knockdown may result from apoptosis, necroptosis, or autophagic cell death. To clarify the specific reasons, we conducted additional experiments in MHCC97H-shCDON cells treated with ferroptosis inhibitor Fer-1, autophagy inhibitor 3-MA, pan-caspase inhibitor Z-VAD-FMK (apoptosis), and necroptosis inhibitor Necrostatin-1 (Nec-1). As shown in Figure 4H, only Fer-1 significantly rescued the proliferation defect induced by CDON knockdown, while the other inhibitors showed no notable rescue effect. Furthermore, Western blot analysis demonstrated that CDON knockdown did not appreciably alter the expression levels of cleaved caspase-3 (an executioner caspase indicative of apoptosis), RIPK3, MLKL, or phosphorylated MLKL at Ser358 (markers of necroptosis activation) (Figure 4I). Treatment with Z-VAD-FMK or Nec-1 did not substantially affect these markers. This might be due to the relatively low basal levels of apoptosis and necrosis in HCC cells. Pharmacological inhibition of these pathways would not necessarily alter the expression levels of their signature proteins in the absence of overt pathway activation. Regarding autophagy, CDON knockdown alone did not significantly affect the LC3B-II/LC3B-I ratio or p62 levels, suggesting that autophagic flux remains unaltered upon CDON deficiency. Notably, however, 3-MA treatment efficiently decreased the LC3B-II/I ratio and increased p62 levels in MHCC97H-shCDON cells, confirming that the autophagy inhibitor was functionally active in our experimental system. By contrast, SLC7A11, a key negative regulator of ferroptosis, was markedly downregulated upon CDON knockdown, and this reduction was partially reversed by Fer-1 treatment, whereas the other inhibitors exerted no such effect. These results collectively indicate that CDON deficiency-induced proliferation suppression and lipid peroxidation are primarily mediated by ferroptosis, rather than apoptosis, necroptosis, or autophagic cell death. The above results suggest that the stiff ECM promotes the expression of CDON, thereby inhibiting ferroptosis in HCC cells.

### CDON inhibits p53 expression in HCC cells through HMGB1

To investigate the regulatory mechanism by which stiff ECM promotes HCC ferroptosis through CDON, we isolated the co-immunoprecipitation products of CDON in Huh7-LVCDON-Flag cells, and then identified the proteins bound to CDON (absolute log2 fold change (log2FC) > 5) using mass spectrometry and bioinformatics analysis. We intersected the immunoprecipitation-mass spectrometry (IP-MS) results with ferroptosis-related genes from the FerrDb V2 database, and the results indicated that CDON might interact with HMGB1 (Figure 5A). Co-immunoprecipitation (Co-IP) results indicated that CDON and HMGB1 interacted with each other (Figure 5B). The immunofluorescence results in Huh7-LVCDON-Flag cells showed that CDON and HMGB1 were mainly co-localized in the cytoplasm (Figure 5C). Glutathione S-transferase (GST) Pull-Down Assays indicated that HMGB1 and CDON have a direct binding relationship (Figure 5D).

Studies have shown that knockdown of HMGB1 can inhibit ferroptosis in vascular endothelial cells 30. The HMGB1/ p53 complex affects the cytoplasmic localization of its binding partners. Knocking out HMGB1 in cells can promote the entry of p53 from the nucleus into the cytoplasm 31, 32. In the cell nucleus, p53 can promote ferroptosis by directly inhibiting the transcription of SLC7A11^33^. These findings suggest that decreased expression of HMGB1 reduces the nuclear levels of p53 by promoting its export from the nucleus, thereby weakening the transcriptional repression of SLC7A11 and inhibiting ferroptosis in cells. Next, we investigated the effect of CDON on the subcellular distribution of HMGB1 and p53 between the cytoplasm and nucleus. We isolated cytoplasmic and nuclear proteins from Huh7-LVCDON-Flag cells and analyzed the expression levels of CDON, HMGB1, and p53 in both compartments using Western blot assays. The results demonstrated that CDON reduced the expression of HMGB1 and p53 in both the cytoplasm and nucleus (Figure 5E). Co-IP experiments in Huh7 cells revealed that HMGB1 binds to p53 (Figure 5F). Previous studies have shown that HMGB1 facilitates the binding of p53 to DNA 34. The enhanced interaction between HMGB1 and p53 stabilizes the p53 protein and decreases its ubiquitination-mediated degradation 35. Does CDON affect the binding of HMGB1 and p53? We performed Co-IP assays on Huh7-LVCDON-Flag cells. The results showed that CDON reduced the expression levels of HMGB1 and p53 in these cells and weakened the interaction between HMGB1 and p53 (Figure 5G). To investigate whether CDON promotes the degradation of HMGB1 and p53, HCC cells were treated with a protein biosynthesis inhibitor, CHX (10 μM), at the indicated time points. These experiments showed that both knockdown and overexpression of CDON influenced HMGB1 and p53 degradation (Figure 5H-I). Building on this observation, to clarify the specific degradation pathway involved, Huh7-LVCDON cells were treated with the proteasome inhibitor MG132 (10 μM, 8 h). Western blot analysis revealed that CDON promoted p53 degradation via the proteasome pathway (Figure 5J). To further explore the mechanism, Co-IP was employed to assess the impact of CDON on the ubiquitination level of p53. The results demonstrated that CDON promoted P53 ubiquitination (Figure 5K). Next, to examine how manipulations of CDON and HMGB1, and treatment with Leptomycin B (LMB, a nuclear export inhibitor targeting CRM1), affect p53 mRNA, Huh7 HCC cells were treated with LVCDON, LVHMGB1, siHMGB1, or LMB (20 nM, 24 h), either alone or in combination. RNA was extracted from the treated groups and subjected to qRT-PCR. The results showed that changes in CDON and HMGB1 expression, as well as LMB treatment, did not affect p53 mRNA levels (Figure 5L). Subsequently, total proteins were extracted, and cytoplasmic and nuclear proteins were separated using a cytoplasmic and nuclear protein extraction kit, followed by a Western blot. The findings demonstrated that CDON could reduce HMGB1 expression in both the cytoplasm and nucleus. Consequently, the binding of HMGB1 and p53 in the nucleus was disrupted, promoting the release of p53 into the cytoplasm through the CRM1-dependent nuclear export pathway. This led to decreased p53 expression in both the nucleus and the cytoplasm (Figure 5M).

The above research results suggest that the binding of CDON and HMGB1 in the cytoplasm can reduce the expression of HMGB1 in the cytoplasm and nucleus, weaken the binding of HMGB1 and p53 in the nucleus, and promote the release of p53 into the cytoplasm through the CRM1-dependent nuclear export pathway and its degradation via the ubiquitin-proteasome pathway.

### CDON inhibits ferroptosis by regulating the expression of SLC7A11

We established a stable cell line by transfecting MHCC97H-shCDON cells with an SLC7A11-overexpressing virus, followed by Western blot assays and measurements of ferroptosis-related indicators, including GSH, ROS, MDA, ferrous ions and GPX4 specific activity. The results demonstrated that CDON knockdown decreased the expression of SLC7A11, GPX4, FTH1, and GPX4 specific activity and increased the expression of LOX and ACSL4, leading to substantial GSH depletion, elevated ROS and MDA levels, and significant accumulation of ferrous ions. Conversely, overexpression of SLC7A11 reversed these ferroptosis-related effects (Figures S4A, 6A, 6C, and 6G). Next, we transfected Huh7-LVCDON-Flag cells with siSLC7A11. Western blot analyses and assessments of ferroptosis markers (GSH, ROS, MDA, ferrous ions, GPX4 specific activity) revealed that CDON overexpression enhanced SLC7A11, GPX4, and FTH1 expression and GPX4 specific activity, decreased the expression of LOX and ACSL4, increased GSH levels, reduced ROS and MDA levels, and decreased ferrous ion accumulation. In contrast, SLC7A11 knockdown intensified ferroptosis-related changes (Figures S4B, 6B, 6D, and 6G). The results from the C11-BODIPY staining assay, which detects lipid peroxidation, showed that knockdown of CDON increased lipid peroxidation levels in HCC cells. Conversely, overexpression of SLC7A11 reduced the lipid peroxidation. Overexpression of CDON alleviated lipid peroxidation, whereas knockdown of SLC7A11 induced lipid peroxidation in HCC cells (Figures 6E and 6F). The above results suggest that CDON inhibits ferroptosis by regulating the expression of SLC7A11.

### CDON promotes the progression of HCC by regulating the expression of SLC7A11

To investigate the impact of CDON on the progression of HCC following the inhibition of ferroptosis through the upregulation of SLC7A11 expression, we performed colony formation assays and CCK-8 assays in MHCC97H-shCDON-LVSLC7A11 stable cell lines and Huh7-LVCDON-Flag cells transfected with siSLC7A11. The results indicated that CDON influences the proliferative capacity of HCC cells by modulating SLC7A11 expression (Figure 7A-B). Additionally, wound healing assays and Transwell migration and invasion assays were conducted on these treated cells. The findings demonstrated that CDON regulates the migratory and invasive abilities of HCC cells through SLC7A11 expression (Figure 7C-D).

We conducted subcutaneous tumor formation experiments in nude mice using the MHCC97H-shCDON-LVSLC7A11 stable cell lines. The *in vivo* imaging results indicated that CDON affects the tumor growth by regulating the expression of SLC7A11(Figure 7E-H). In summary, CDON promotes liver cancer progression *in vitro* and *in vivo* by regulating SLC7A11 expression.

### CDON promotes the resistance of HCC to sorafenib by regulating ferroptosis

We treated Huh7-LVCDON cells with the sorafenib (5 μM) for 24 hours. Western blot analyses and assessments of ferroptosis markers (GSH, ROS, MDA, ferrous ions) revealed that CDON overexpression enhanced SLC7A11, GPX4, and FTH1 expression, increased GSH levels, reduced ROS and MDA levels, and decreased LOX and ACSL4 expression and ferrous ion accumulation. In contrast, sorafenib treatment intensified ferroptosis-related changes (Figures S4C and 8A-B). CDON inhibits ferroptosis in HCC cells via SLC7A11, whereas sorafenib attenuates CDON's inhibitory effect on ferroptosis by suppressing SLC7A11.

Subsequent colony formation assays and CCK-8 assays indicated that CDON promoted the proliferative capacity of HCC cells, while sorafenib treatment reduced the proliferation ability of HCC cells. (Figure S5A-B). Additionally, wound healing assays and Transwell migration and invasion assays were conducted on these treated cells. The findings demonstrated that CDON promoted the migratory and invasive abilities of HCC cells, and sorafenib inhibited those abilities of HCC cells (Figure S5C-D). MHCC97H-shCDON and Huh7-LVCDON cells were treated with sorafenib in a series of concentration gradients (0.1, 2.5, 5, 10, 20, 40 μM) for 24 hours. Cell viability was detected by CCK-8. The results showed that the Half-maximal inhibitory concentration (IC50) for sorafenib in Huh7-LVCDON cells was higher than that of the control group (LVcontrol IC50=4.814 μM, LVCDON IC50=6.956 μM), and in MHCC97H-shCDON cells was lower than that of the control group (shcontrol IC50=7.808 μM, shCDON IC50=5.853 μM) (Figure 8C). CDON promotes the resistance of HCC to sorafenib.

We used CDON knockdown and sorafenib in combination in an HCC orthotopic transplantation model. The results showed that both CDON knockdown and sorafenib treatment inhibited tumor growth, with the combination treatment producing a more significant inhibitory effect (Figure 8D-E). Furthermore, both treatments also suppressed lung metastasis of liver cancer (Figure 8F). The levels of GSH and MDA in mouse liver cancer tissues were detected. The results showed that sorafenib reduced GSH levels and increased MDA levels. CDON knockdown alone also reduced GSH and increased MDA, and the combination of CDON knockdown and sorafenib further intensified these ferroptosis-related changes (Figure 8G). We also conducted immunohistochemical staining on the liver tissues of mice. The results showed that the expression of the proliferation marker protein ki67 decreased in both the sorafenib and CDON knockdown groups, and the decrease was more significant in the combined group of the two. The ferroptosis-related protein GPX4 also showed similar results. Although studies have shown that sorafenib also plays an important role in the polarization of immune macrophages and angiogenesis in liver cancer 36, there was no significant trend observed for the macrophage marker proteins CD68 and F4/80, as well as the angiogenesis marker protein CD31. Considering the particularity of the immune deficiency in nude mice.

In summary, CDON can not only promote the resistance of liver cancer cells to sorafenib by regulating ferroptosis, but also jointly regulate tumor progression with sorafenib.

### The stiff ECM promotes the translocation of YAP1 into the nucleus by integrins, where it binds to FOXA1, thereby transcriptionally regulating the expression of CDON

How does the stiff ECM regulate ferroptosis through the CDON-SLC7A11 pathway? Numerous studies have shown that the integrin used as a mechanical sensor is mainly a heterodimer formed by the β1 subunit and various α subunits (such as α5 and αV), which senses changes in the stiffness of the extracellular matrix and regulates transcriptional co-activator YAP1 dephosphorylation and nuclear translocation through pathways such as the Hippo signaling pathway 37. Once in the nucleus, YAP1 binds to transcription factors such as TEAD4, targeting downstream molecules and exerting various effects, including the promotion of tumor progression 38-40. Interestingly, in most cases, YAP1 is expressed in both the cytoplasm and the nucleus, but it only functions in the nucleus 41. In the Hippo pathway, the activation of Mst1/2 kinases successively phosphorylates Lats1/2 kinases and YAP1. The activated Lats kinase mainly phosphorylates YAP1 (pYAP1) at the Ser127 site (other phosphorylation sites including T63, S61, S109, S138, S164, S289, S351, S381 and S384) and promotes the retention of YAP1 in the cytoplasm 41.

After culturing MHCC97H and Huh7 cells on ECM of varying stiffness for 72 hours, we extracted proteins from the cytoplasm and nucleus using a commercial kit and measured the expression levels of YAP1 and pYAP1. The results demonstrated that in ECM with high stiffness, both the phosphorylation and total protein levels of YAP1 in the cytoplasm decreased, while the nuclear expression of YAP1 increased (Figure S6A). These findings suggest that a stiff ECM promotes YAP1 dephosphorylation and its translocation into the nucleus, where it functions as a transcriptional co-activator. We used GEPIA to conduct correlation analysis between CDON and key molecules involved in the signal transduction of ECM stiffness, including integrin β1 (ITGB1), integrin β3 (ITGB3), integrin α-5 (ITGA5), integrin α-v (ITGAV), and YAP1. Among them, there was a strong correlation between CDON and YAP1, followed by CDON and ITGB (Figure S6B). The HCC cells may sense the changes in the stiffness of the ECM through integrin β1, and regulate the expression of CDON through YAP1. Therefore, we transfected MHCC97H and Huh7 cells with YAP1 siRNA and overexpression plasmids, respectively. Western blot analysis showed that CDON expression increased in YAP1-overexpressing cells and decreased in YAP1-knockdown cells (Figure 9A), indicating that YAP1 regulates CDON expression. We treated HCC cells with different concentration gradients (0, 10, 50, 100, 200, 400 μM) of ATN-161, a novel α5β1 antagonist. Western blot results showed that the expression of CDON decreased as the concentration of ATN-161 increased. Beyond 100 μM, the expression of CDON decreased and reached a plateau (Figure S6C). Then, we treated HCC cells with ATN-161 (100 μM, 24 hours) and used the cytoplasmic and nuclear separation kit to separate cytoplasmic and nuclear proteins. The results showed that after ATN-161 treatment, the expression of CDON in the cytoplasm decreased, while the levels of YAP1 protein and its phosphorylation increased, and the level of YAP1 protein in the nucleus decreased (Figure 9B). The nuclear translocation of YAP1 was weakened. After treatment with ATN-161 and overexpression of YAP1 in HCC cells, the Western blot results indicated that integrins promoted the expression of CDON by regulating the nuclear translocation of YAP1 (Figure 9C).

Which transcription factor targeting CDON binds to YAP1 to exert transcriptional co-activation and thereby regulate CDON expression? We used three transcription factor databases—UCSC, Cistrome DB, and hTFtarget—to predict transcription factors targeting CDON and identified their intersection. The results showed that FOXA1, SOX2, KLF5, and SP1 might be the transcription factors targeting CDON (Figure 9D). We analyzed the interactions among FOXA1, SOX2, KLF5, and YAP1 using STRING, which revealed that these proteins interact with each other (Figure 9E). Next, we examined the correlation between FOXA1, SOX2, KLF5, and CDON using GEPIA. The results indicated that FOXA1 had the highest and most significant correlation with CDON (R = 0.39, *P* = 5.3 × 10^-15^) (Figure 9F). We then investigated whether FOXA1 expression, like CDON, varies with ECM stiffness. Western blot assays showed that FOXA1 expression was elevated in MHCC97H and Huh7 HCC cells cultured on stiff ECM (Figure 9G). We assessed the expression of FOXA1, CDON, and SLC7A11 in MHCC97H cells after siRNA-mediated FOXA1 knockdown and in Huh7 cells after FOXA1 overexpression by Western blotting. The results demonstrated that FOXA1 overexpression increased CDON and SLC7A11 levels, whereas FOXA1 knockdown decreased their expression (Figure 9H), suggesting that FOXA1 regulates CDON and SLC7A11 expression. Co-immunoprecipitation (Co-IP) experiments revealed that YAP1 and FOXA1 physically interact in MHCC97H and Huh7 cells (Figure 9I). Using JASPAR, we predicted FOXA1 binding sites within the CDON promoter region, selecting six sites with scores of 8 or higher. Chromatin immunoprecipitation (ChIP) assays (Figure 9J) and site-specific deletion followed by dual-luciferase reporter assays (Figure 9K) in control and FOXA1-overexpressing MHCC97H cells indicated that FOXA1 regulates CDON expression by binding specifically to the third site of the CDON promoter.

MHCC97H cells and Huh7 cells were respectively transfected with FOXA1 siRNA and overexpression plasmids. Total protein extracts were prepared, and cytoplasmic and nuclear proteins were isolated. The Western blot results indicated that FOXA1 influenced the nuclear translocation of YAP1 (Figure 9L). The immunofluorescence assay showed similar results (Figure 9M). Does the stiffness of the ECM affect the binding of FOXA1 and YAP1? MHCC97H HCC cells were cultured on soft or stiff ECM for 72 h, with or without siFOXA1 or integrin inhibitor ATN-161 treatment. Cell lysates were subjected to Co-IP using anti-FOXA1 antibodies, followed by immunoblotting with the indicated antibodies. The results demonstrated that stiff ECM significantly enhanced the interaction between FOXA1 and YAP1 compared to soft ECM (Figure 9N). Integrin blockade significantly reduced FOXA1 protein expression. This stiffness-induced association was attenuated by integrin blockade. Thus, integrin signaling promotes FOXA1-YAP1 complex assembly through dual mechanisms: maintaining FOXA1 expression and facilitating YAP1 nuclear translocation. Knockdown of FOXA1 under stiff ECM conditions abolished the YAP1-FOXA1 interaction, confirming that FOXA1 is essential for the stiffness-promoted complex formation. These data collectively establish that ECM stiffness actively promotes the assembly of the FOXA1-YAP1 transcriptional complex through integrin signaling, and that FOXA1 serves as an indispensable component for this mechanically induced protein complex.

Multiple studies have shown that CTGF is a direct target of YAP1 and TEAD 42, 43. Therefore, we chose CTGF as the positive control. We conducted ChIP-qPCR, and the results showed that FOXA1 did not bind to the CTGF promoter region (Figure 9O). YAP1 bound to the CTGF promoter region, and knocking down FOXA1 did not affect its signal. YAP1 binding to CTGF is not dependent on FOXA1. FOXA1 bound to site 3 of the CDON promoter region, and the signal significantly weakened after knocking down FOXA1. The results of YAP1 were similar. YAP1 bound to CDON in a FOXA1-dependent manner.

However, it remains unclear whether FOXA1 requires YAP1 for its stability or nuclear localization in a stiff ECM. HCC cells were treated with a protein biosynthesis inhibitor, CHX (10 μM), under the indicated conditions. These experiments showed that FOXA1 does not require YAP1 for its stability under either soft or stiff conditions (Figure S6D). We knocked down YAP1 in a stiff ECM. Immunofluorescence staining showed that the FOXA1 expression increased in stiff ECM, and nuclear localization of FOXA1 did not show any significant change after knocking down YAP1 in the stiff ECM (Figure S6E). This indicates that FOXA1 is intrinsically stable and does not rely on YAP1 for its nuclear residence.

The schematic diagram showed that a stiff ECM promotes dephosphorylation of YAP1 by integrins, facilitating its nuclear translocation, where it binds to the transcription factor FOXA1 (Figure 9P). This complex targets the CDON promoter region, thereby enhancing CDON expression. Mechanistically, CDON primarily binds to HMGB1 in the cytoplasm, reducing HMGB1 levels in both the cytoplasm and nucleus, which weakens the binding of HMGB1 and p53 in the nucleus and promotes the release of p53 into the cytoplasm through the CRM1-dependent nuclear export pathway and its degradation via the ubiquitin-proteasome pathway. Decreased p53 expression promotes SLC7A11 expression and regulates ferroptosis in HCC cells by alleviating the transcriptional repression of SLC7A11. Furthermore, CDON promotes sorafenib resistance in liver cancer cells.

## Discussion

In the present study, we observed that tumor cells pre-cultured on stiff polyacrylamide (PA) substrates exhibited enhanced colony formation and migration, even after being detached and replated on conventional tissue culture plastic. This raises a critical question: do the mechanical priming effects persist after the removal of the original stiffness cue, and if so, for how long? Our findings are consistent with the emerging concept of mechanical memory, wherein cells retain phenotypic and molecular adaptations induced by past physical microenvironments, even after subsequent culture on substrates with different mechanical properties 44-46. In line with this, previous studies have shown that fibroblasts and mesenchymal stem cells cultured on stiff substrates maintained altered contractility, gene expression, and chromatin remodeling for days to weeks after transfer to soft matrices 47, 48. Cancer cells have also been reported to retain stiffness-induced invasive and migratory phenotypes through sustained nuclear translocation of mechanosensitive transcription factors such as YAP1 and RUNX2, as well as persistent chromatin accessibility changes 49, 50. A review suggested that biophysical adaptations favourable for metastasis are retained via mechanical memory 51. Importantly, the duration of mechanical memory appears to depend on both the magnitude and the duration of the initial mechanical stimulus, with longer priming times (e.g., ≥7 days) and higher stiffness thresholds (>8 kPa) leading to more stable epigenetic alterations, including histone acetylation, DNA methylation, and non-coding RNA regulation 48, 52. NFATC2, as a transcription factor, is required for memory acquisition and enhanced confined migration 53. However, the current study does not systematically track the decay kinetics of these memory effects, and future time-course experiments using reversible hydrogel systems will be essential to delineate the half-life and reversibility of mechanical memory in cancer cells. Nevertheless, our results support the notion that mechanical priming in the primary tumor microenvironment may leave a lasting imprint on tumor cell behavior, which could influence subsequent metastatic steps even after cells have left the original niche. This concept has profound implications for the design of mechano-based therapeutic strategies, as targeting persistent epigenetic modifications or downstream mechanotransduction pathways may help erase detrimental mechanical memory and reduce metastatic potential.

Traditional treatment strategies primarily target the cancer cells themselves; however, their efficacy is often limited and susceptible to drug resistance. This underscores the urgent need to deepen our understanding of the underlying mechanisms driving liver cancer progression 54. ECM stiffness significantly influences the progression of various tumors, including liver cancer, and contributes to chemotherapy resistance 55, 56. ECM stiffness influences the expression of F-actin around mitochondria by regulating the nuclear factors Spire1C and Arp2/3, as well as mitochondrial fission mediated by DRP1 and MIEF1/2. These changes in mitochondrial dynamics can alter mitochondrial reactive oxygen species (ROS) production and NRF2-driven antioxidant transcriptional responses, including cystine uptake and glutathione metabolism. Ultimately, this affects the antioxidant stress resistance of cancer cells and their sensitivity to ROS-dependent chemotherapy drugs 57. The stiffness of the ECM acts as an initiating factor by promoting the activation of Piezo1 and calcium ion influx via the integrin β1/miR-625-5p pathway. This activation inhibits the ubiquitination of HIF-1α, thereby facilitating the expression of angiogenic factors and accelerating angiogenesis in HCC. Notably, the stiff ECM also enhances the expression of COL1 through the same integrin β1/miR-625-5p pathway, leading to the formation of an even stiffer ECM. This creates a positive feedback loop that exacerbates HCC angiogenesis and promotes tumor metastasis 58. Studies have shown that a stiff ECM can enhance the stability of FTL mRNA by regulating the intracellular SMYD3/H3K4me3/PD-L2 pathway, thereby inhibiting SLC7A11-mediated ferroptosis in HCC cells. The deletion of PD-L2, combined with sorafenib and anti-PD-1 antibody treatment, significantly sensitizes HCC cells and inhibits tumor growth *in vivo* 24. However, the specific molecular mechanism by which ECM stiffness regulates HCC progression and chemotherapy resistance remains to be fully elucidated. Our study demonstrates that increased ECM stiffness promotes the dephosphorylation of the transcriptional co-activator YAP1 by integrins, facilitating its nuclear translocation and interaction with the transcription factor FOXA1. This interaction transcriptionally upregulates the expression of CDON. CDON primarily binds to HMGB1 in the cytoplasm, reducing HMGB1 levels in both the cytoplasm and nucleus, which weakens the binding of HMGB1 and P53 in the nucleus and promotes the release of p53 into the cytoplasm through the CRM1-dependent nuclear export pathway and its degradation via the ubiquitin-proteasome pathway. Ultimately, this regulatory cascade modulates SLC7A11-mediated ferroptosis and tumor progression in HCC cells by diminishing p53's transcriptional repression of SLC7A11. Furthermore, CDON attenuates sorafenib-induced ferroptosis and mitigates the drug's inhibitory effects on HCC cell proliferation, migration, and invasion, and promotes sorafenib resistance in liver cancer cells. Inhibition of CDON in combination with sorafenib results in a more potent anti-tumor effect *in vivo*.

Our research reveals a novel mechanism regulating ferroptosis and tumor progression in HCC cells. These findings indicate that ECM stiffness-induced CDON plays a critical role in SLC7A11-mediated ferroptosis and sorafenib resistance through the HMGB1/p53 pathway. Consistent with previous studies 58-60, these findings also emphasize that ECM stiffening is essential for driving HCC progression and chemotherapy resistance. Previous research has shown that CDON mainly functions as a receptor in the Hedgehog (Hh) signaling pathway and also participates in the WNT signaling pathway, playing roles in embryonic development, vascular smooth muscle calcification, and other processes 61-65. Interestingly, regardless of the Hh or WNT signaling pathways, their main function is to relieve the inhibition of downstream proteins. Our study is the first to show that CDON affects the expression of p53 and its binding to HMGB1 by regulating HMGB1, thus relieving the inhibition of p53 on SLC7A11. This implies that CDON also plays an important role in cancer. The absence of CDON expression can lead to reduced activation and/or mislocalization of integrin β1 12. ECM stiffness drives cancer chemotherapy resistance or angiogenesis through the integrin β1-pMLC-YAP1 pathway or integrin-phosphorylated PXN in focal adhesions, which activates Rac1-YAP1 signaling 13, 14. Combined with our study, this suggests that CDON is not only transcriptionally regulated by YAP1-FOXA1 complex but may also affect YAP1 nuclear entry by influencing integrinβ1. This may potentially form a positive feedback loop of integrin β1-YAP1-CDON-integrin β1. However, when we examined the integrins in MHCC97H-shCDON liver cancer cells, as well as their activated forms and the downstream YAP1 and its phosphorylation levels, the results showed that CDON knockdown had no significant effect on the integrins and their downstream pathway (Figure S6F). Therefore, our data does not support the existence of such a positive feedback loop in HCC. This might be due to the significant differences in the signaling networks between satellite cells in the muscles and liver cancer cells. Taken together, these results indicate that the stiffness-integrin-YAP1-FOXA1-CDON axis operates predominantly in a unidirectional manner in HCC, rather than forming a feedback loop through integrin β1. Therefore, investigating the specific molecular mechanisms of CDON in the regulation of tumor proliferation and invasion by ECM stiffness is of great clinical significance for predicting sorafenib resistance and tumor progression in patients.

Our research reveals a novel regulatory role of the FOXA1-YAP1 complex in ECM stiffness. FOXA1 is a pioneer transcription factor that establishes gene expression capacity and plays a central role in biological processes such as organogenesis, differentiation, glycolipid metabolism, proliferation, migration, invasion, and drug resistance 66. We found that FOXA1 expression is upregulated in a stiff ECM, consistent with previous studies showing that ECM stiffness promotes FOXA1 expression via the DAB2IP/PI3K/AKT pathway, thereby regulating the growth of colon cancer stem cells 67. As a transcriptional co-activator, YAP1 can integrate mechanical signals from the ECM (such as stiffness and shear force) with epigenetic modifications (such as m6A methylation) to regulate various biological functions, including proliferation, metastasis, ferroptosis, immune escape, and metabolism, leading to treatment resistance and disease progression 68. Matrix mechanics can induce the dephosphorylation of YAP1 and its entry into the nucleus to bind with multiple transcription factors such as BACH1, promoting disease progression 69. FOXA1 not only physically interacts with the transcriptional co-activator YAP1—which undergoes dephosphorylation and nuclear translocation in response to stiff ECM signals—but also directly binds to the promoter region of CDON to transcriptionally upregulate its expression. This coordinated action establishes a mechanically responsive transcriptional complex that links extracellular stiffness to downstream gene regulation. Importantly, this is consistent with previous reports demonstrating that ECM stiffness can regulate FOXA1 expression through integrin-mediated signaling pathways 67, further supporting the notion that mechanical cues actively modulate the availability of key transcription factors to execute stiffness-dependent transcriptional programs. Functionally, the YAP1-FOXA1-CDON axis promotes HCC progression and, importantly, inhibits ferroptosis in HCC cells through the HMGB1/p53-SLC7A11 pathway. Our findings indicate that CDON plays a crucial role in regulating ferroptosis resistance in HCC cells in response to ECM stiffness. CDON regulates the key ferroptosis defense system by promoting the expression of SLC7A11, maintaining the redox homeostasis of cells. This function enables cancer cells with high CDON expression to survive in a stressed microenvironment. Therefore, CDON constitutes a key molecular bridge between ECM stiffness and ferroptosis sensitivity, and its expression and that of its upstream regulator YAP1 may serve as biomarkers for predicting the response of HCC patients to ferroptosis-inducing therapy.

Despite the robust *in vitro* and *in vivo* evidence demonstrating that CDON promotes sorafenib resistance through the HMGB1/p53-SLC7A11/ferroptosis pathway, we acknowledge several limitations. Most importantly, we lack matched local clinical cohorts with complete sorafenib treatment and follow-up data; therefore, whether CDON expression in HCC patients correlates with clinical response to sorafenib remains unknown. Additionally, our *in vivo* experiments were performed in immunodeficient nude mice, precluding assessment of immune microenvironment contributions, and the decay kinetics of stiffness-induced mechanical memory were not examined.

To address these gaps, we plan to initiate a multicenter prospective observational study enrolling newly diagnosed advanced HCC patients scheduled for first-line sorafenib therapy, with CDON expression assessed on pretreatment biopsies and rigorous longitudinal follow-up to correlate expression levels with progression-free and overall survival. Public pharmacogenomic databases will also be explored for further validation. These efforts are expected to commence within 12 months. Until then, our findings remain mechanism-based and hypothesis-generating, requiring clinical corroboration before translational application. In the future, inhibitors targeting CDON can be designed and combined with sorafenib for the treatment of HCC patients.

## Materials and Methods

### Patient samples and cell culture

The human materials used in this study were all approved by the Ethics Committee of Tongji Hospital (TJ-IRB20220820). 32 pairs of liver cancer tissues and adjacent tissues from patients who underwent liver cancer surgery at Wuhan Tongji Hospital were collected. Human liver cancer cell lines (MHCC97H, PLC/PRF/5, SNU-398, Huh7), normal human liver cells (THLE2 and THLE3), as well as HEK-293T cells and Hep1-6 cells were purchased from the Cell Bank of the Chinese Academy of Sciences (Shanghai). In an appropriate culture medium, 10% fetal bovine serum (FBS; New Zealand) was added, and the cells were cultured at 37°C with 5% CO_2_. Research Resource Identifiers (RRIDs) of cell lines are shown in Table S1 (Supporting Information).

### Cell transfection

We used Lipofectamine™3000 (Invitrogen) to transfect siRNA at a cell density of 30-50%, and transfected plasmids at a cell density of 60%-80%. In this study, the siRNAs, plasmids and viruses used were as follows: YAP1 siRNA and SLC7A11 overexpression plasmid (Tsingke Biotech, Beijing, China); SLC7A11 siRNA (Hippo Biotech, Huzhou, China); FOXA1 siRNA, Renilla luciferase reference plasmid, CDON promoter wild-type and single-point deletion mutant plasmid (Obio Technology, Shanghai, China); YAP1 overexpression plasmid, backbone plasmid pSPAX2 and capsid plasmid pMD2.G (Genomeditech, Shanghai, China); FOXA1 overexpression plasmid, CDON knockdown plasmid and CDON overexpression lentivirus (Genechem Technology, Shanghai, China). Among them, the SLC7A11 overexpression plasmid and the CDON knockdown plasmid were packaged as lentiviruses for use. We transfected the target gene lentiviral plasmids (SLC7A11 overexpression plasmid or CDON knockdown plasmid) and pMD2.G and pSPAX2 into HEK-293T cells using transfection reagents (PEI, MedChemExpress). The lentiviruses were harvested on the 4th day. The viruses were filtered through a 0.45 μm filter and stored at -80°C. To construct stable cell lines, the lentiviruses were used for multiple transfections of HCC cells. The target sequences of the knocked-down molecules are shown in Table S3 (Supporting Information). The knockdown efficiency of YAP1, FOXA1, and SLC7A11 is shown in Figure S7.

### Preparation of polyacrylamide hydrogel

The glass coverslips or glass dishes were treated with 0.1M sodium hydroxide, followed by treatment with APES (Solarbio, A7440) and 0.5% glutaraldehyde solution (Sigma). Then it is air-dried in the dark. 40% acrylamide and 2% methylene bisacrylamide (Sigma) are mixed in ddH_2_O at a specific ratio as shown in Table S4 (Supporting Information), and 10% ammonium persulfate (volume ratio 1/100, Solarbio) and TEMED (volume ratio 1/1000, Solarbio) are added to promote gel polymerization. Then the gel mixture is dropped onto the DMDCS (Sigma-Aldrich) treated cover glass, and the gel is covered with a 6-well plate slide. The gel is washed with PBS and 2 mg/ml sulfosuccinimide SANPAH (Thermo) is added. Then the gel is exposed to ultraviolet light for 30 minutes to activate the crosslinking agent and incubated at 37°C overnight with 0.1mg/ml type I collagen (50mmol/L HEPES Solution). The excess collagen is washed away with PBS, and ultraviolet irradiation is performed for 30 minutes in a clean bench, followed by inoculation of cells. The specific procedures were performed according to the method described by Tse J *et al*. and Caliari S *et al*. 70, 71. In this study, the extracellular matrix with a Young's modulus of 0.48 ± 0.16 kPa and 10.61 kPa was respectively referred to as the soft and stiff extracellular matrix.

### Western blot assays

According to the instructions, the cell nucleus and cytoplasm protein extraction kit (Beyotime, Shanghai, China) was used to extract the cell nucleus and cytoplasm proteins. The methods of protein preparation and immunoblotting have been described in previous studies 72. The primary antibodies are shown in Table S5 (Supporting Information).

Mouse orthotopic xenograft HCC model and orthotopic xenograft HCC model in mouse with liver fibrosis.

BALB/c nude and C57BL/6 mice (male, 6 weeks old, Jicuiyaokang Biological Technology, Nanjing, China) were housed under standard conditions. All animal experiment procedures were conducted under the approval of the Ethics Committee of Tongji Hospital, Tongji Medical College, Huazhong University of Science and Technology (TJH-202306033).

The stable transfected MHCC97H or Huh7 liver cancer cells, as well as the control liver cancer cell lines, were constructed using viruses. VitroGel® 3D (TWG001) is unmodified synthetic hydrogel without toxic or inflammatory responses. The product manual indicates that there are no issues related to biocompatibility and safety, and it is suitable for *in vivo* experiments. These cells were mixed with different stiffness levels of hydrogels (formed from the original solution VitroGel and dilutions at 1:0 and 1:3, resulting in two different hardness levels of 12 kPa and 0.6 - 1.5 kPa) 26. Mouse orthotopic xenograft HCC models were established by injection of cell suspension (50 μl cell suspension, with a cell volume of 2 × 10^6^ per mouse). The mice were monitored every two days, and bioluminescence imaging was performed weekly using IVIS Lumina K Series III. The image brightness values were normalized using Living image (Perkinelmer). Five weeks later, the mice were sacrificed and the liver and lung tissues were taken for living image. General pictures were taken, and some liver tissues were frozen and fixed. The lung tissue sections were fixed, embedded, and stained with HE.

Carbon tetrachloride and olive oil (in a ratio of 1:3) were intraperitoneally injected twice a week (5 μl/g per mouse according to the mouse's body weight). After 8 weeks, one mouse from each group was randomly selected and sacrificed to obtain the liver for HE and Masson staining to assess the degree of liver fibrosis. Subsequently, a mouse orthotopic xenograft HCC model was constructed by injecting Hep1-6 (mouse HCC) cells (50 μl cell suspension, with 2 × 10^6^ cells per mouse). The mice were monitored every two days, and after three weeks, they were sacrificed and the liver was photographed.

### Wound healing assay

Place the 2-well cell culture insert in a 6-well plate. Add 80 µl of cell suspension to each well, with a concentration of 4 × 10^5^/ml. After the cells adhere, remove the 2-well culture insert and wash each well twice with PBS. Cultivate the cells in DMEM. Take pictures using an Olympus microscope at 0 and 24 hours after removing the culture insert.

### Detection of GSH, MDA, ROS, ferrous ions, and GPX4 specific activity

Using the GSH assay kit (A006-2-1, Nanjing Jiancheng, China), MDA assay kit (BC0025, Solarbio, Beijing, China), ROS assay kit (S0033S; Beyotime Biotechnology, China), ferrous ion assay kit (E-BC-K881-M, Elabscience, Wuhan, China), and GPX4 specific activity kit (E-BC-K883-M, Elabscience, Wuhan, China). The levels of GSH, MDA, ROS, ferrous ions, and GPX4 specific activity in the cells were detected according to the instructions of the corresponding kits.

### Chromatin immunoprecipitation quantitative PCR (ChIP-qPCR)

MHCC97H cells transfected with the FOXA1 overexpression plasmid or siRNA were fixed with formaldehyde. The detection was carried out in accordance with the instructions of the ChIP Kit (Cell Signaling Technology, #9003). In addition to the reagents in the kit, the following additional antibody was used Table S5 (Supporting Information). DNA was quantified via qPCR using primers in Table S7 (Supporting Information).

### Dual-luciferase reporter assays

After transfecting the target plasmid and the Renilla luciferase reference plasmid (in a ratio of 5:1) into the cells for 72 hours, the detection was carried out according to the instructions of the Dual Luciferase Assay Kit (DL101-01, Vazyme, Nanjing, China).

### Co-immunoprecipitation (Co-IP)

Cells were lysed in ice-cold NP40 buffer supplemented with protease and phosphatase inhibitors for 90 min on ice. After centrifugation at 12,000 × g for 15 min at 4°C, the supernatant was collected, and protein concentration was determined using a BCA assay (Servicebio, Wuhan, China). For each immunoprecipitation reaction, total protein lysate was pre-cleared with 20 µL of Protein A/G agarose beads (Promoter, Wuhan, China). The pre-cleared lysate was then incubated with 2 µg of antibody overnight at 4°C with gentle rotation. The next day, 30 µL of fresh Protein A/G beads were added to each sample and incubated for another 2 h at 4°C. The beads were collected by brief centrifugation and washed with ice-cold lysis buffer. Bound proteins were eluted by boiling the beads in loading buffer at 95°C for 10 min. The obtained proteins were then subjected to Western blot. The antibodies are shown in Table S5 (Supporting Information).

### Glutathione S-transferase (GST) pull-down assays

GST-HMGB1 and His-CDON proteins were purified by Obio Technology (Shanghai, China). Glutathione Magnetic agarose beads (MCE) were washed three times with 500 µL of washing buffer (PBS: 140 mM NaCl, 2.7 mM KCl, 10 mM Na2HPO4, 1.8 mM KH2PO4, pH 7.4). The beads were then incubated with 10-20 µg of purified GST-HMGB1 in 500 µL of washing buffer at room temperature for 30 min under gentle rotation. After magnetic separation, the supernatant was discarded, and the beads were washed three times with 1 mL of washing buffer to remove unbound proteins. Subsequently, the GST-HMGB1-immobilized beads were incubated with 10-20 µg of purified His-CDON in 500 µL of washing buffer at room temperature for 30 min with rotation. Following incubation, the beads were magnetically separated. The beads were then washed three times with 1 mL of washing buffer to eliminate non-specifically bound proteins. Bound proteins were eluted by incubating the beads with 3-5 bead volumes of elution buffer (10 mM reduced glutathione in 50mM Tris-HCl, pH 8.0) at room temperature for 10 min with gentle mixing. The eluates were collected by magnetic separation. The samples were boiled in loading buffer and loaded onto SDS-PAGE gels for Western blot.

### Statistical analysis

Image analysis was performed using ImageJ software, and statistical data analysis was conducted using GraphPad Prism 8.0 software. All data were recorded as mean ± SEM. *P* < 0.05 was considered statistically significant. For comparisons between two groups, after confirming normality through the Shapiro-Wilk test and homogeneity of variance through the F test, the Student's t-test was applied. For comparisons among multiple groups, the Shapiro-Wilk test was used to confirm normality, and the Brown-Forsythe test was used to confirm homogeneity of variance, after which one-way analysis of variance was employed. The Kaplan-Meier curve was analyzed using the log-rank test. Analysis of independent prognostic risk factors was conducted using univariate and multivariate Cox regression analysis.

Other materials and methods applied in this research are available in the Supporting Information.

## Supplementary Material

Supplementary methods, figures and tables.

## Figures and Tables

**Figure 1:**
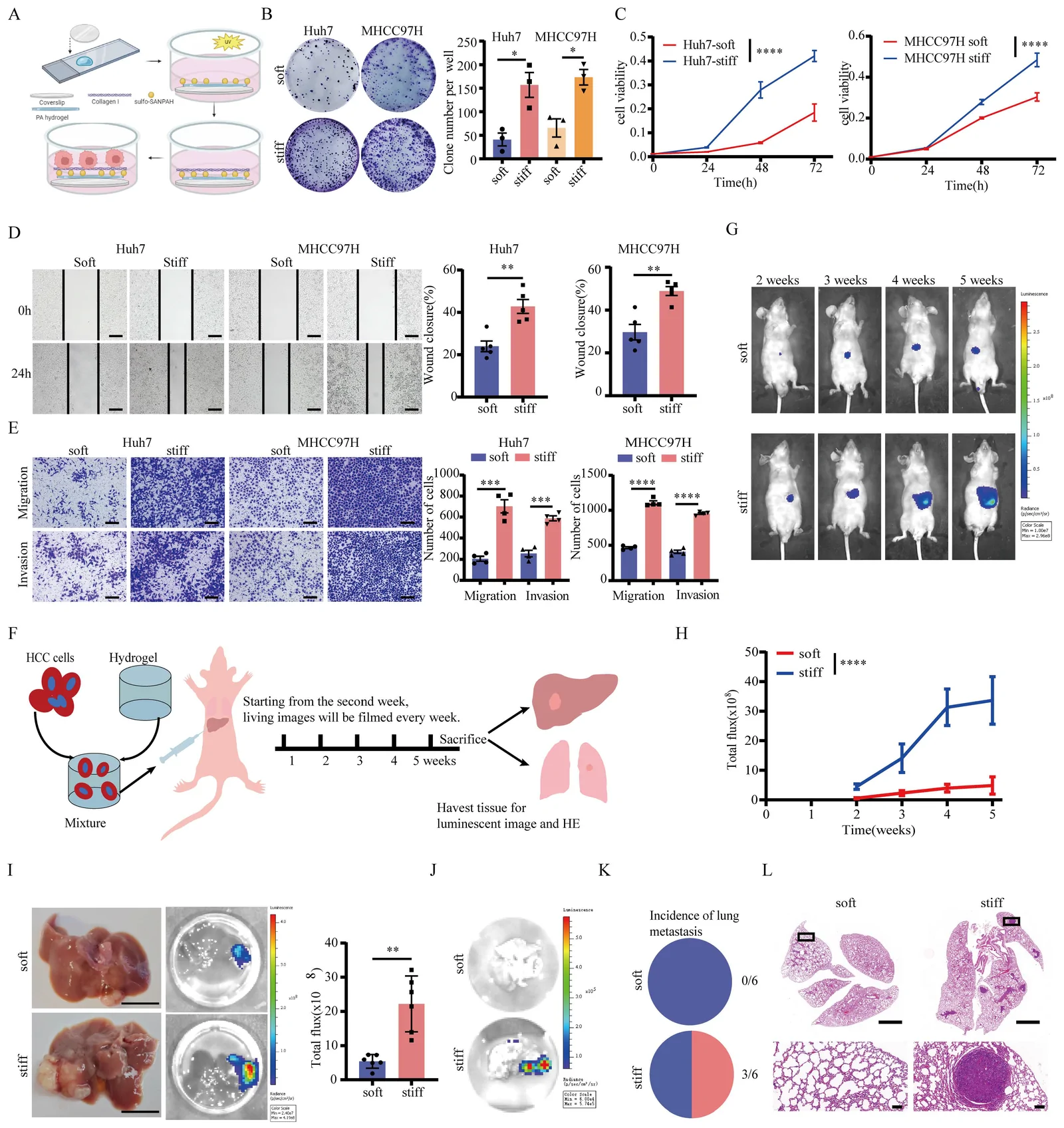
** Stiff ECM promotes the progression of liver cancer.** A Schematic diagram of the preparation of polyacrylamide hydrogel. B Representative images and statistical graphs of colony formation assays after Huh7 and MHCC97H cells are cultured on soft and stiff polyacrylamide hydrogels (*n*=3). C Statistical graph of CCK-8 assay (*n*=3). D-E Representative images and statistical graphs of wound healing assay (*n*=5) and Transwell migration and invasion assay (*n*=4). Scale bar: 100 µm. F Schematic diagram of the orthotopic xenograft HCC model in mice with different extracellular matrix stiffness. G-H Representative bioluminescent images and statistical graphs of bioluminescent signals of mice with different extracellular matrix stiffness at 2 - 5 weeks (*n*=6). I Representative images of livers, bioluminescent images of livers, and statistical graphs of bioluminescent signals taken in mice treated with different extracellular matrix stiffness. Scale bar: 10 mm. J Representative bioluminescent images of lungs of mice with different extracellular matrix stiffness. K Incidence of lung metastasis of tumors in mice with different extracellular matrix stiffness. L The incidence of lung metastasis of tumors and lung HE staining images in mice treated with different extracellular matrix stiffness. Scale bar: 2mm (up), 100 µm (down).

**Figure 2:**
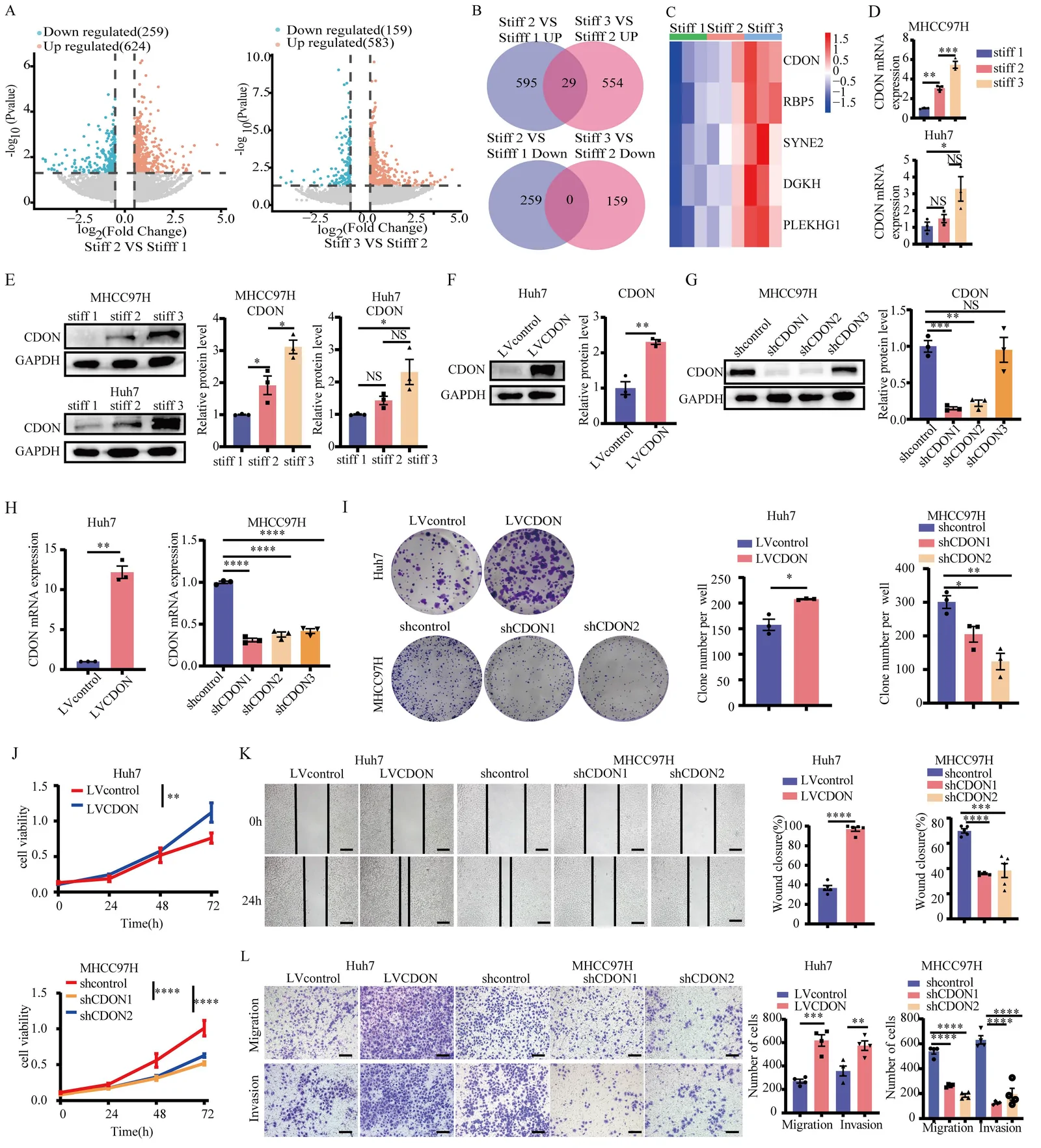
** CDON is overexpressed in stiff ECM and promotes the progression of HCC *in vitro*.** A Volcano plot of differential expression analysis of transcriptome data of MHCC97H cells cultured on three extracellular matrices with increasing stiffness (absolute log2 fold change (log2FC) > 0.5). Stiff 1, Stiff 2, and Stiff 3 are 0.48 ± 0.16 kPa, 2.01 ± 0.75 kPa, and 10.61 kPa, respectively. B Venn diagram of molecules with increasing and decreasing expression levels as the ECM stiffness increases. C Heat map of the expression of five molecules related to the prognosis of patients with liver cancer among 29 molecules whose expression increases with the increase of extracellular matrix stiffness. D mRNA levels of CDON in MHCC97H and Huh7 cells detected by qRT - PCR (*n*=3). E Western blot was used to detect the protein expression of CDON in MHCC97H and Huh7 cells under different extracellular matrix stiffness, and statistical analysis was performed (*n*=3). F-G Western blot was used to detect the protein level of CDON in the constructed Huh7 - LVCDON and MHCC97H - shCDON HCC cell lines, and statistical analysis was performed (*n*=3). H qRT - PCR was used to detect the mRNA levels of CDON in the constructed MHCC97H - shCDON and Huh7-LVCDON liver cancer cell lines. I-J Colony formation assay and CCK-8 were used to detect the proliferation ability of MHCC97H cells with CDON knockdown and Huh7 liver cancer cell lines with CDON overexpression. K Wound healing assay was used to detect the migration ability of MHCC97H - shCDON and Huh7-LVCDON liver cancer cell lines. Scale bar: 100 µm. L Transwell migration and invasion assay was used to detect the invasion and metastasis ability of MHCC97H - shCDON and Huh7 - LVCDON liver cancer cell lines. Scale bar: 100 µm.

**Figure 3:**
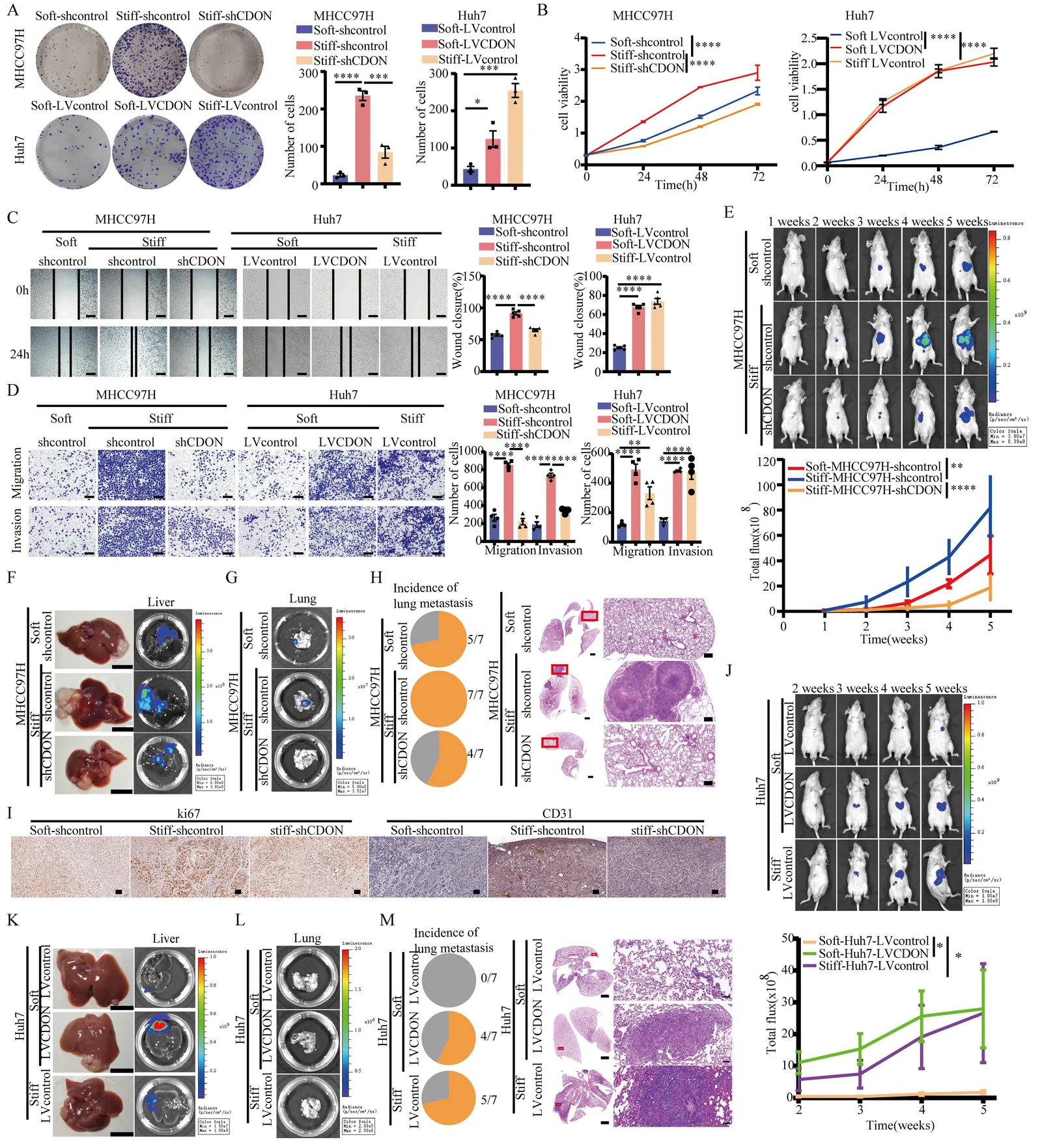
** Stiff ECM promotes the progression of HCC by regulating the expression of CDON.** A-B Colony formation and CCK-8 assay were used to detect the proliferation ability of MHCC97H and Huh7 HCC cells treated as shown in the figure (*n*=3). C Wound healing assay was used to detect the migration ability of MHCC97H and Huh7 HCC cells treated as shown in the figure. Scale bar: 100 µm (*n*=5). D The Transwell migration and invasion assay was used to detect the invasion and migration ability of MHCC97H and Huh7 liver cancer cells, as shown in the figure. Scale bar: 100 µm (*n*=3). E After mixing control and CDON-knockdown MHCC97H cells with hydrogels of different stiffness and performing an orthotopic xenograft HCC model in mice, bioluminescent images of mice were continuously acquired for five weeks. Representative images and statistical graphs (*n*=7). F Representative images of livers and bioluminescent images of livers. Scale bar: 10 mm. G Representative bioluminescent images of lungs. H The incidence of lung metastasis of tumors and HE staining of the lungs. Scale bar: 1mm (left), 200 µm (right). I Detection of ki67 and CD31 protein levels in the liver tumor of mice by immunohistochemical staining. Scale bar: 50 µm. J Representative bioluminescent images and statistical graphs of the orthotopic xenograft HCC model in mice with indicated Huh7 cells were shown (*n*=7). K Representative images of livers and bioluminescent images of livers. Scale bar: 10 mm. L Representative bioluminescent images of lungs. M The incidence of lung metastasis of tumors and HE staining of the lungs in mice with indicated Huh7 cells were shown. Scale bar: 2mm (left), 100 µm (right).

**Figure 4:**
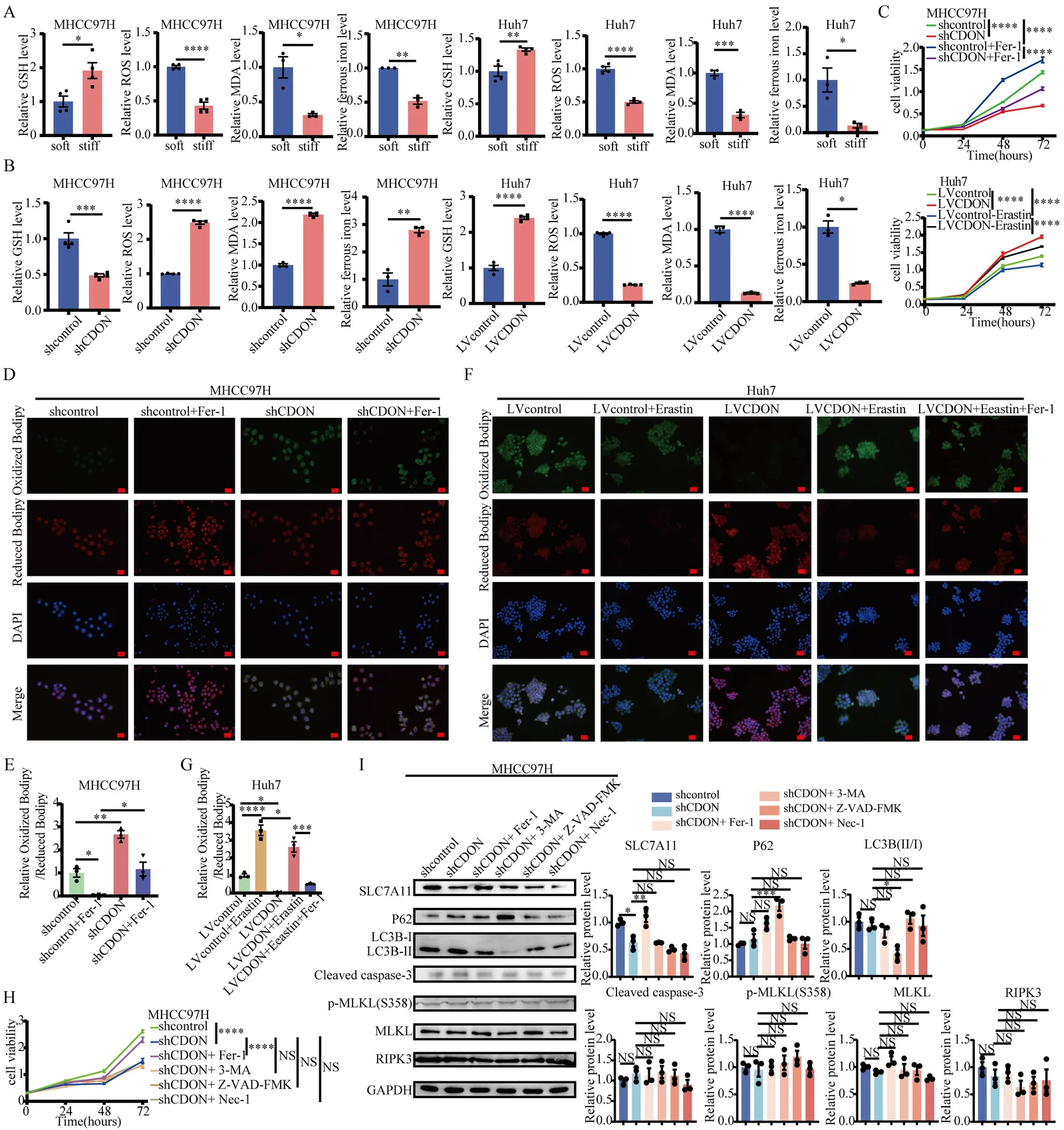
** CDON inhibits ferroptosis in HCC cells.** A Levels of GSH, ROS, MDA, and ferrous ions in MHCC97H and Huh7 cells cultured on soft and stiff ECM. The levels of GSH and ROS were determined in quadruplicate (*n*=4), while MDA and ferrous iron contents were measured in triplicate (*n*=3). B Levels of GSH, ROS, MDA, and ferrous ions in CDON-overexpressing Huh7 cells and CDON-knockdown MHCC97H cells. C MHCC97H-shCDON cells were treated with the ferroptosis-specific inhibitor Ferrostatin-1 (Fer-1). Huh7-LVCDON HCC cell lines were treated with the ferroptosis inducer Erastin. CCK-8 assays were used to detect the proliferation ability of cells subjected to the above treatments (*n*=3). D-G The C11-BODIPY staining showed the lipid peroxidation level of the indicated cells (*n*=4). H MHCC97H-shCDON cells were treated with Fer-1 (ferroptosis inhibitor), 3-MA (autophagy inhibitor), Z-VAD-FMK (pan-caspase inhibitor), and Necrostatin-1 (Nec-1, necroptosis inhibitor). CCK-8 was used to detect the changes in cell viability in different treatment groups. I Western blot analysis was performed to detect the expression levels of LC3B-II/I, P62, cleaved caspase-3, RIPK3, p-MLKL (Ser358), MLKL, SLC7A11, and GAPDH (as a loading control) across the different treatment groups.

**Figure 5:**
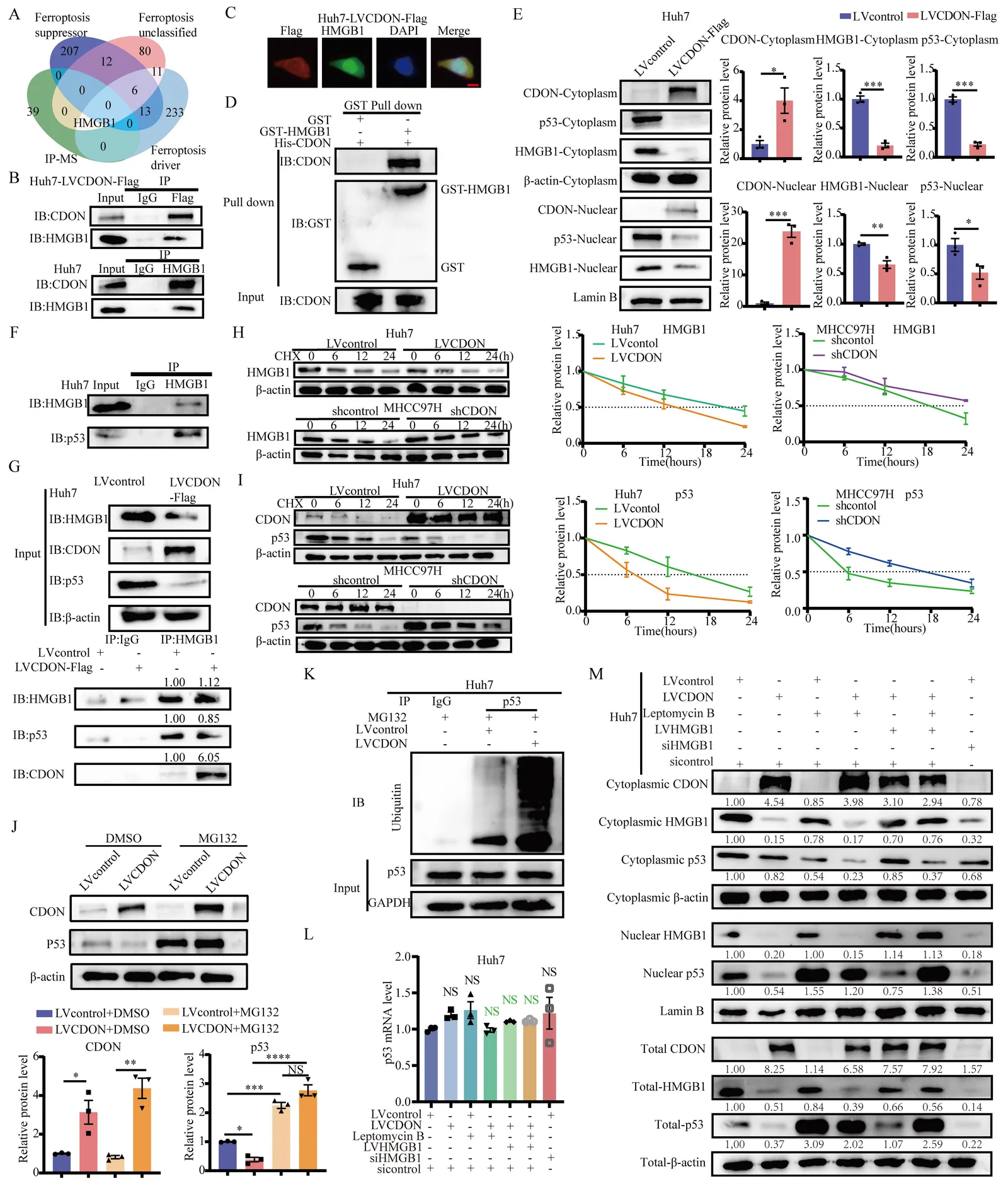
** CDON inhibits p53 expression in HCC cells through HMGB1.** A Venn diagram of the intersection between proteins in the IP-MS results of Huh7 -LVCDON-Flag cells and molecules in the ferroptosis database FerrDb V2. B Co-IP diagram of CDON and HMGB1 proteins. C Immunofluorescence diagram of CDON and HMGB1 in Huh7-LVCDON-Flag cells. D Glutathione S-transferase (GST) Pull-Down Assays of GST-HMGB1 and His-CDON (*n*=3). E Western blot was used to detect the protein levels of CDON, HMGB1, and p53 in the cytoplasm and nucleus of control and Huh7-LVCDON-Flag cells, and a statistical graph was made (*n*=3). F Co-IP assay was used to detect the protein interaction between HMGB1 and p53 in Huh7 cells. G Co-IP assay was performed on control and Huh7-LVCDON -Flag cells to detect the effect of CDON overexpression on the protein interaction between HMGB1 and P53. H-I Huh7-LVCDON and MHCC97H-shCDON HCC cells were treated with a protein biosynthesis inhibitor (CHX, 10 μM) for the indicated times. Protein levels were analyzed by Western blot using the indicated antibodies. The intensity of each band was quantified, normalized to the control, and plotted relative to the initial time point (0 h) (*n*=3). J Huh7-LVCDON cells were treated with MG132 (10 μM) for 8 h. Western blot analysis was used to detect the protein levels of CDON and P53 (*n*=3). K Huh7-LVCDON cells were treated with MG132 (10 μM) for 8 h to accumulate ubiquitinated proteins. Cell lysates were immunoprecipitated with anti-P53 antibody or control IgG, followed by Western blot analysis with anti-Ubiquitin antibody to detect polyubiquitination of p53 (*n*=3). L Huh7 HCC cell line was treated with a combination of LVCDON, LVHMGB1, siHMGB1, and LMB for combinatorial regulation. qRT-PCR detection of the combined regulation effect on the level of p53 mRNA (*n*=3). M Western blot detection of the combined regulation effect on p53 and HMGB1 in the cytoplasm, nucleus, and total protein of cells (*n*=3).

**Figure 6:**
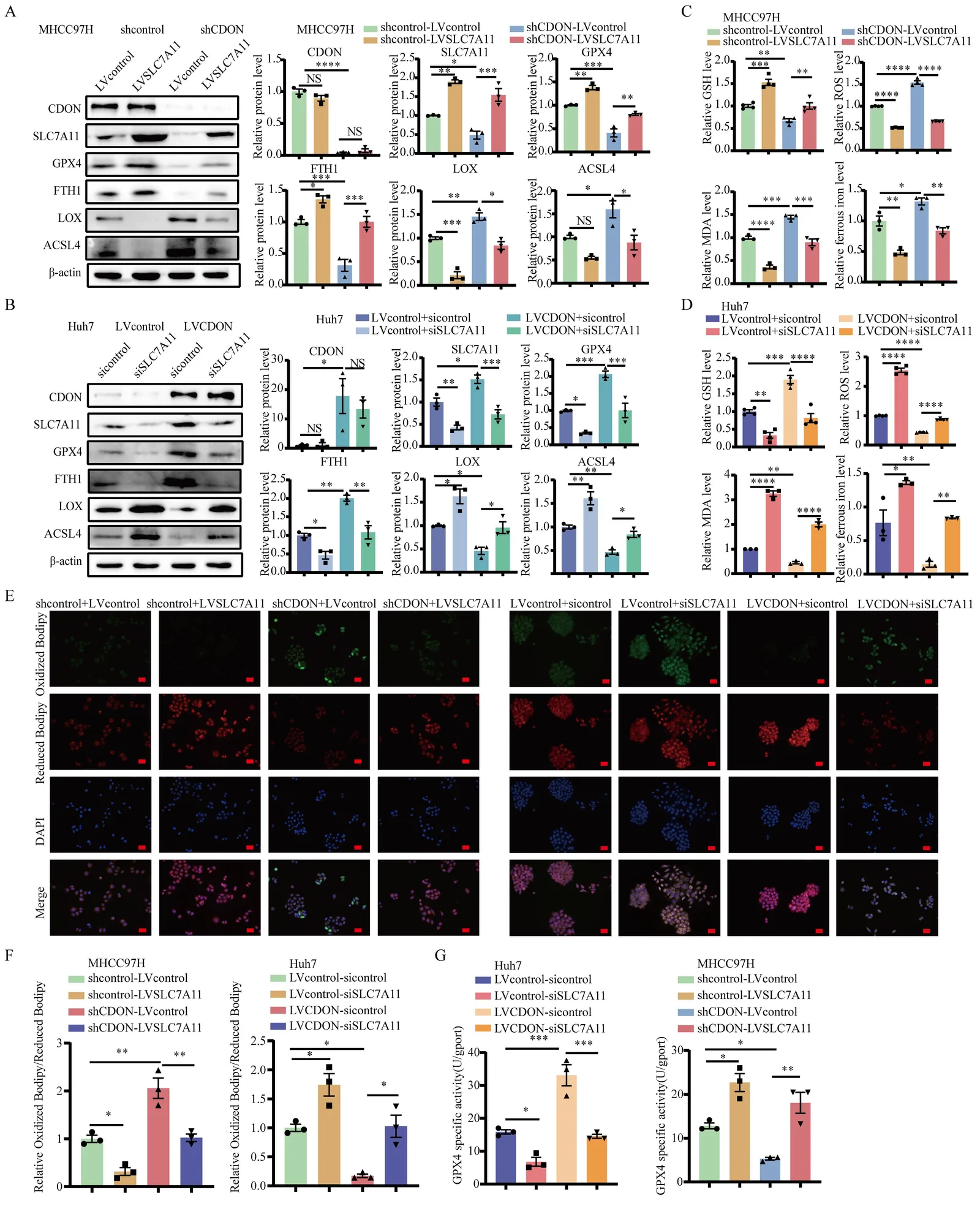
** CDON inhibits ferroptosis by regulating the expression of SLC7A11.** A-B MHCC97H-shCDON cells are transfected with the SLC7A11 overexpression plasmid, and Huh7-LVCDON-Flag cells are transfected with siSLC7A11.Western blot assays and statistical analysis were performed (*n*=3). C-D Statistical graph of the detection of GSH, ROS, MDA, and ferrous ion levels in the indicated cells (*n*=3). E-F The C11-BODIPY staining showed the lipid peroxidation level of the indicated cells (*n*=3). G Statistical graph of the detection of GPX4-specific activity levels in the indicated cells (*n*=3).

**Figure 7:**
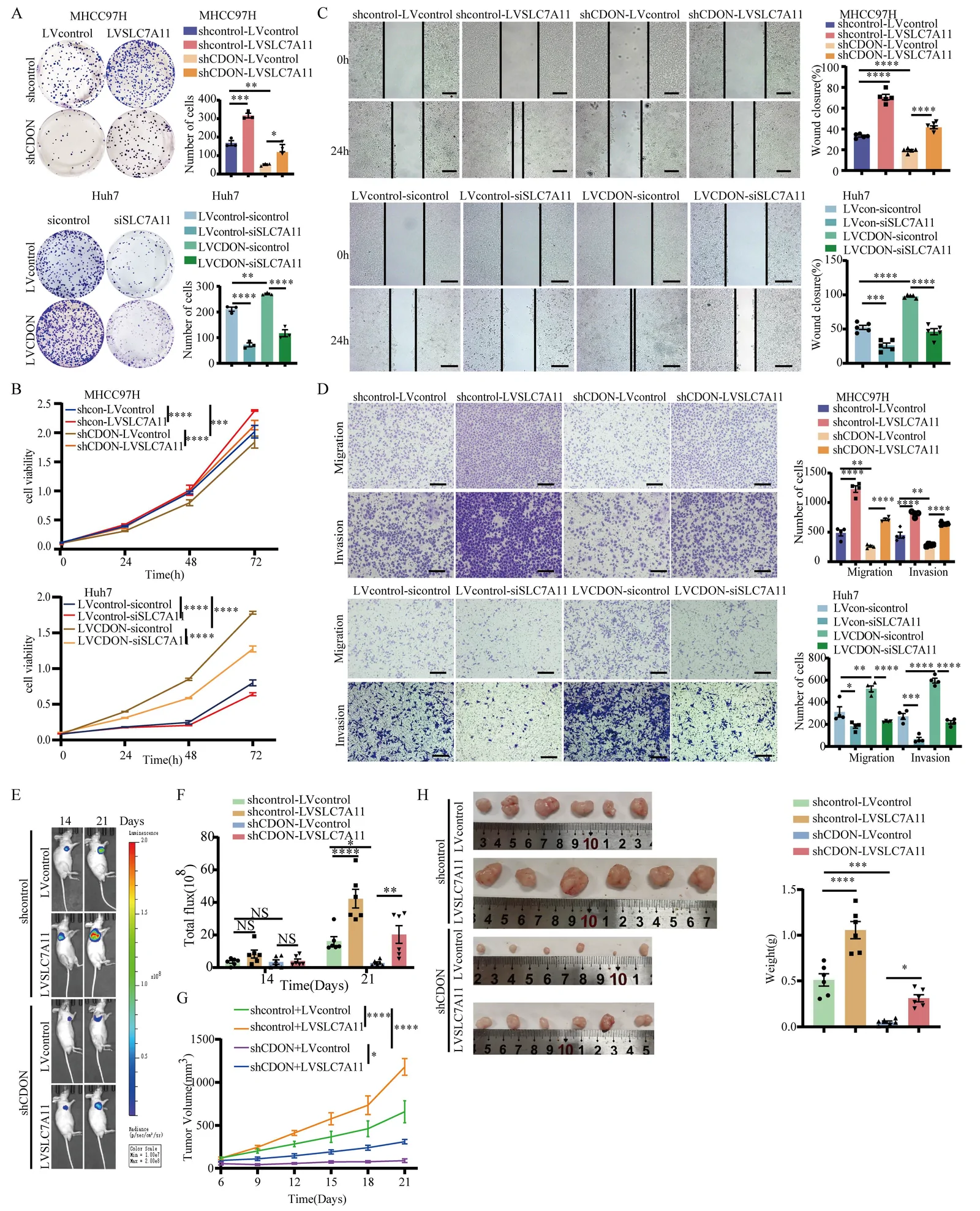
** CDON promotes the progression of HCC by regulating the expression of SLC7A11.** A-B Colony formation and CCK-8 assays were used to detect the proliferation ability of MHCC97H cells with combined regulation of CDON knockdown and SLC7A11 overexpression and Huh7 cells with combined regulation of CDON overexpression and SLC7A11 knockdown (*n*=3). C Wound healing assay was used to detect migration ability in the indicated cells. Scale bar: 100 µm (*n*=5). D The migratory and invasive capacity of the indicated cells was analyzed by Transwell assay. Scale bar: 100 µm (*n*=4). E-F Subcutaneous tumor formation experiments were performed in mice. Bioluminescent images of mice were acquired continuously for two weeks. Representative images and statistical graphs (*n*=6). G Starting from the sixth day until the 21st day, the size of the subcutaneous tumors in the mice was measured every three days for analysis and plotting. H On the 21st day, the mice were sacrificed, and the tumors were isolated, photographed, and weighed.

**Figure 8:**
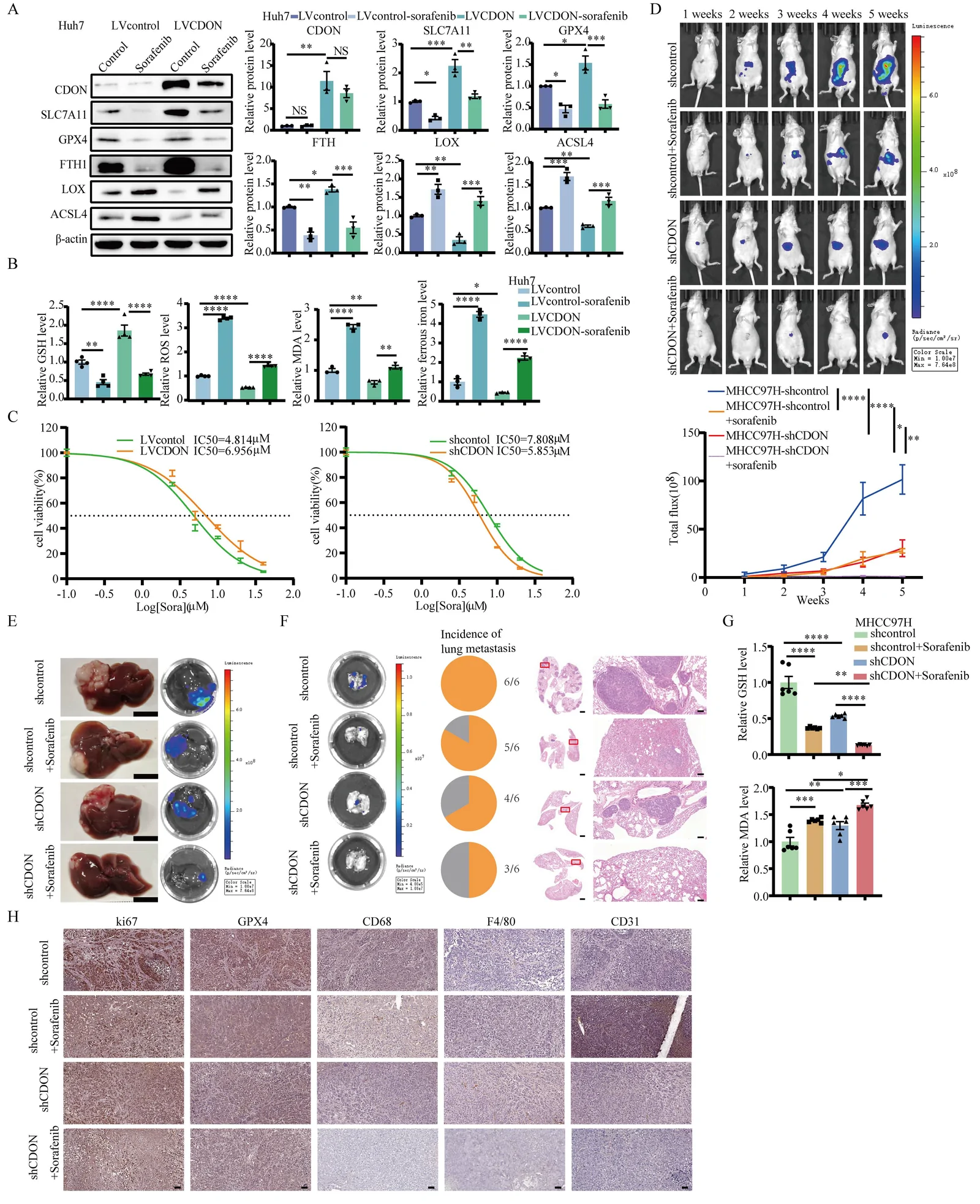
** CDON promotes the resistance of HCC to sorafenib by regulating ferroptosis.** A Huh7-LVCDON-Flag cell is treated with the ferroptosis inducer Sorafenib (5 μM), which inhibits SLC7A11, for 24 hours. Western blot assays and statistical analysis are performed (*n*=3). B Statistical graph of the detection of GSH, ROS, MDA, and ferrous ion levels in the indicated cells. The levels of GSH and ROS were determined in quadruplicate (*n*=4), while MDA and ferrous iron contents were measured in triplicate (*n*=3). C MHCC97H-shCDON and Huh7-LVCDON cells were treated with sorafenib in a series of concentration gradients (0.1, 2.5, 5, 10, 20, 40 μM) for 24 hours. Cell viability was assessed by CCK-8, and the half-maximal inhibitory concentration (IC50) of sorafenib was determined (*n*=3). D Representative bioluminescent images and statistical graphs of the orthotopic xenograft HCC model in mice with indicated MHCC97H cells were shown (*n*=6). E Representative images of livers and bioluminescent images of livers. Scale bar: 10 mm. F Representative bioluminescent images of lungs (left). The incidence of lung metastasis of tumors and HE staining of the lungs in mice with indicated MHCC97H cells were shown (right). Scale bar: 1 mm(left), 100 µm(right). G Statistical graph of the detection of GSH and MDA levels in the liver tumor of mice. H Detection of ki67, GPX4, CD68, F4/80, and CD31 protein levels in the mouse liver by immunohistochemical staining. Scale bar: 50 µm.

**Figure 9:**
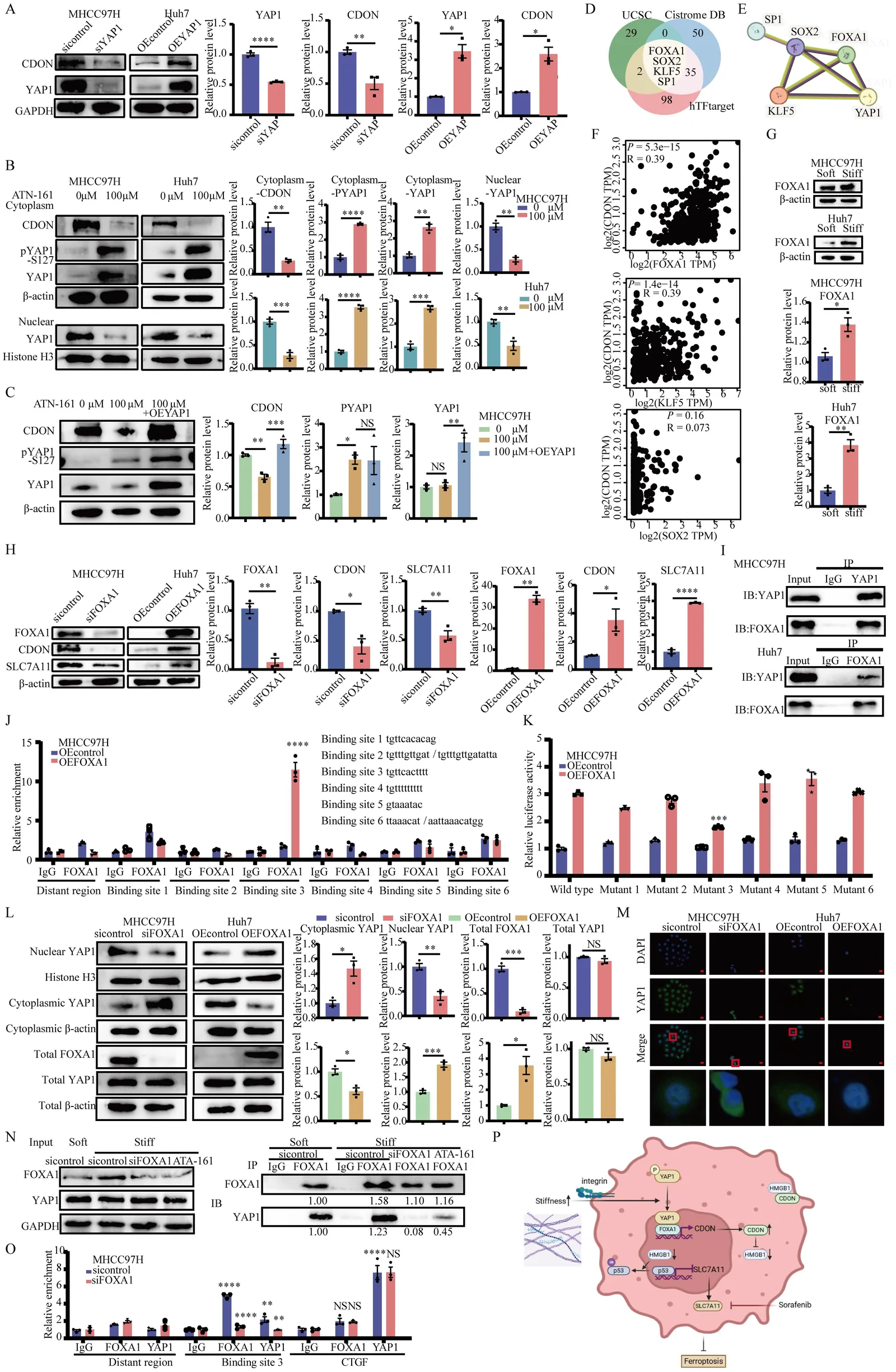
** The stiff ECM promotes the translocation of YAP1 into the nucleus by integrins, where it binds to FOXA1, thereby transcriptionally regulating the expression of CDON.** A Western blot was used to detect the expression of YAP1 and CDON after YAP1 knockdown and overexpression (*n*=3). B Western blot assays were used to detect the expression and distribution of YAP1 and other molecules in MHCC97H and Huh7 cells that were treated with ATN-161, a novel α5β1 antagonist (*n*=3). C Western blot assays were employed to detect the expression levels of YAP1, pYAP1, and CDON in cells under the combined regulation of YAP1 overexpression and ATN-161 (*n*=3). D Venn diagram of the intersection of transcription factors targeting CDON predicted by three databases: UCSC, Cistrome DB, and hTFtarget. E Protein interaction relationship between YAP1 and predicted transcription factors targeting CDON, analyzed by STRING. F Correlation analysis diagram of three transcription factors, FOXA1, KLF5, and SOX2, with CDON by GEPIA. G The expression of FOXA1 was detected by Western blot in MHCC97H and Huh7 cells cultured on soft and stiff ECM conditions (*n*=3). H Western blot was used to detect the expression of FOXA1, CDON, and SLC7A11 in cells with FOXA1 knockdown and overexpression (*n*=3). I Co-IP was used to detect the binding of FOXA1 and YAP1. J Six binding sites with a score of more than eight in the FOXA1-targeted CDON promoter region predicted by JASPAR are selected, and ChIP-qPCR is performed in control and FOXA1-overexpressing MHCC97H cells (*n*=3). K In the indicated cells, a dual-luciferase assay was performed after single-point deletion mutations were made at each of the above six sites (*n*=3). L Western blot assays were used to detect the expression and distribution of YAP1 in cells with FOXA1 knockdown and overexpression (*n*=3). M YAP1 immunofluorescence staining. Scale bar: 20 µm. N Co-IP is used to detect the binding of FOXA1 and YAP1 under the indicated conditions. O ChIP-qPCR is performed in control and FOXA1 knockdown MHCC97H cells (*n*=3). P A schematic diagram illustrating the regulation of ferroptosis in HCC cells by ECM stiffness through CDON. Increased matrix stiffness, acting through integrin signaling, promotes the nuclear translocation of dephosphorylated YAP1 and enhances the binding of the transcription factor FOXA1 in the nucleus, thereby regulating CDON expression. CDON binds to HMGB1, reducing HMGB1 levels in both the cytoplasm and nucleus, which weakens the binding of HMGB1 and p53 in the nucleus and promotes the release of p53 into the cytoplasm through the CRM1-dependent nuclear export pathway and its degradation via the ubiquitin-proteasome pathway. In this way, CDON alleviates the transcriptional repression of SLC7A11 and promotes its expression. This regulation modulates ferroptosis in HCC cells and contributes to resistance to sorafenib.

## Data Availability

All data used and/or analyzed during the current study are available from the corresponding author on reasonable request.
