# Supplementary material for: ECM stiffness inhibits ferroptosis to promote HCC progression via the FOXA1-CDON-HMGB1/p53 pathway
Source: Int J Biol Sci. 2026 Oct 9;22(15):8740–64. doi: 10.7150/ijbs.129038 (PMC13686058; doi:10.7150/ijbs.129038)
Supplement: Supplementary file 1 [file ijbsv22p8740s1.pdf]

Supplementary Figures

Figure S1

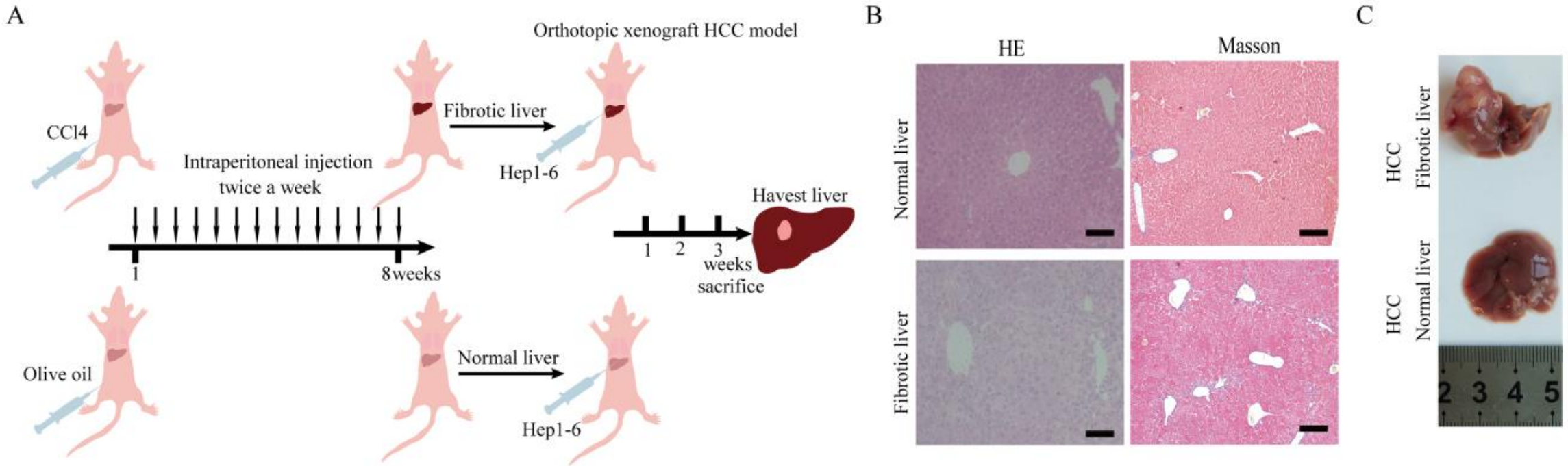


Figure S1 Liver cirrhosis promotes the progression of liver cancer

A Schematic diagram of an orthotopic xenograft hepatocellular carcinoma (HCC) model in mice with liver fibrosis.

B Representative HE and MASSON staining images of livers from cirrhotic and control mice (*n* = 1 mouse per group for histology; 4 mice per group in total). Scale bar: 100 μm.

C Representative images of the liver from mouse orthotopic xenograft HCC mode (*n*=3).

Figure S2

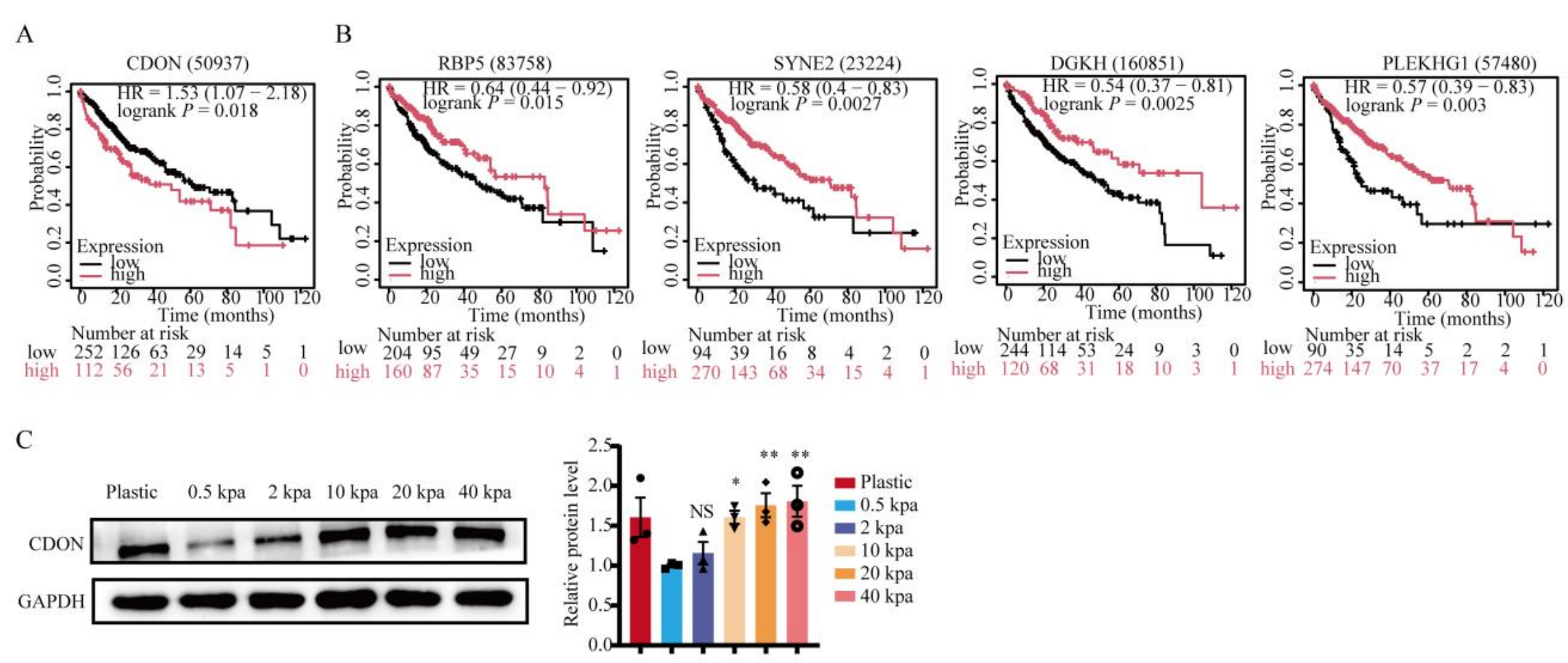


Figure S2 CDON clinical relevance and protein levels

A. Kaplan-Meier (KM) curve of CDON

B. KM curves of molecules (RBP5, SYNE2, DGKH, PLEKHG1)

C Detection of CDON protein levels in different stiffness ECM（tissue culture plastic (TCP, GPa range, representing a supra-physiological stiff reference or positive extreme control), 0.48 ± 0.16 kPa, 2.01±0.75kPa, 10.61 kPa, 19.66 ±1.19 kPa, and 40.40 ± 2.39 kPa) by Western blot. The positive extreme control does not participate in the comparison ($n$=3).

Figure S3

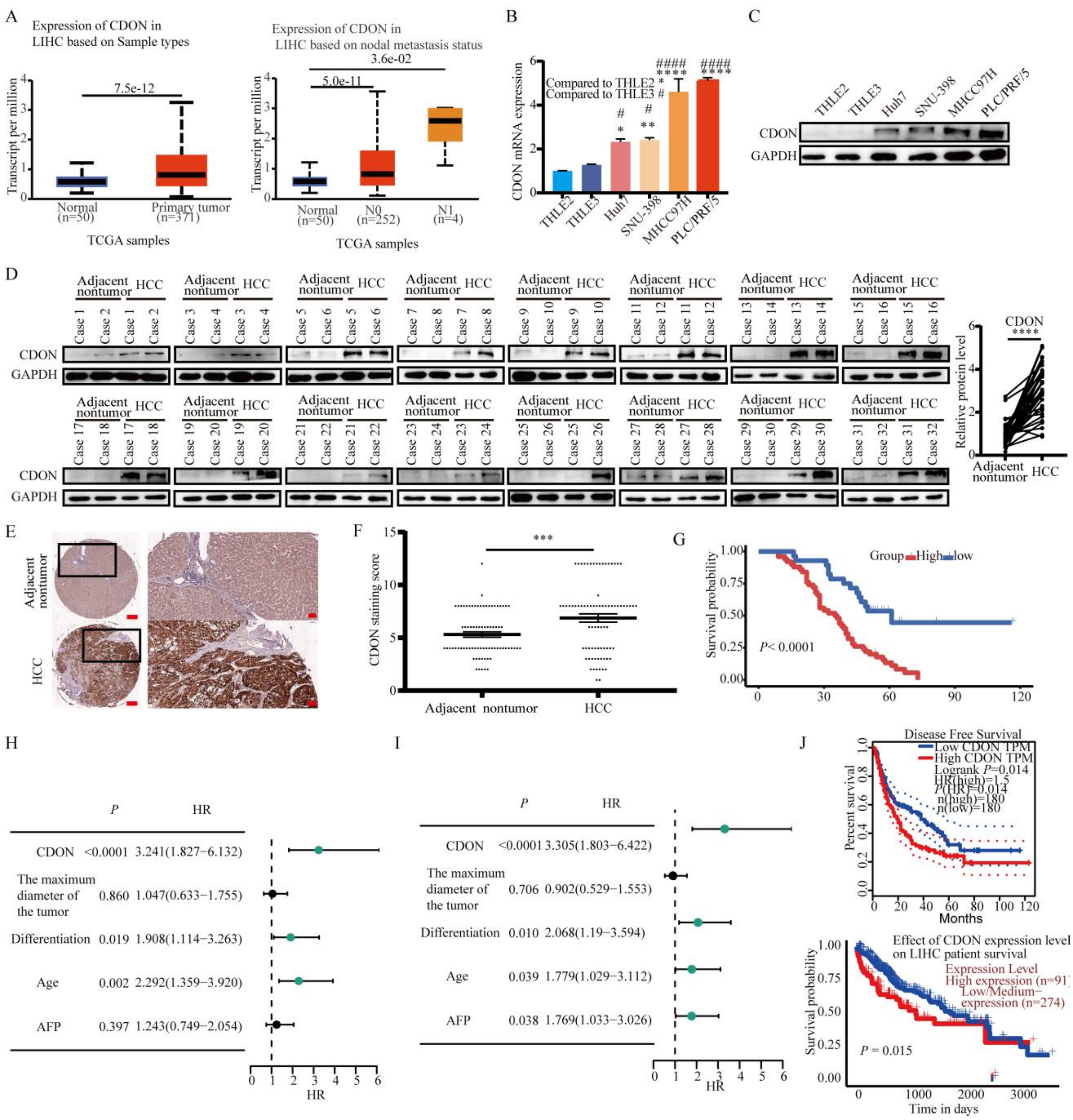


Figure S3 CDON is highly expressed in HCC and serves as an independent prognostic

factor.

A. Expression of CDON in normal individuals and HCC patients, as well as its expression in HCC patients at different stages in The Cancer Genome Atlas (TCGA) data.

B - C. Detection of CDON mRNA and protein levels in normal hepatocytes (THLE2 and THLE2) and HCC cells (Huh7, SNU - 398, MHCC97H, PLC/PRF/5) by quantitative real - time polymerase chain reaction (qRT - PCR) and Western blotting.

D. Detection of CDON protein levels in HCC patients by Western blotting (*n*=32).

E. Detection of CDON protein levels in HCC patients by immunohistochemical staining. Scale bar: 200 µm(left) ,50µm(right).

F. Immunohistochemical score statistics graph (*n* =78).

G The KM curve was plotted by https://www.bioinformatics.com.cn, an online platform for data analysis and visualization.

H Forest Plot of univariate Cox regression analysis of CDON and clinical indicators.

I Forest Plot of multivariate Cox regression analysis.

J Generation of KM and Disease Free Survival curves using GEPIA (http://gepia.cancer - pku.cn/) and Kaplan - Meier plotter (https://kmplot.com/analysis/), respectively.

Figure S4

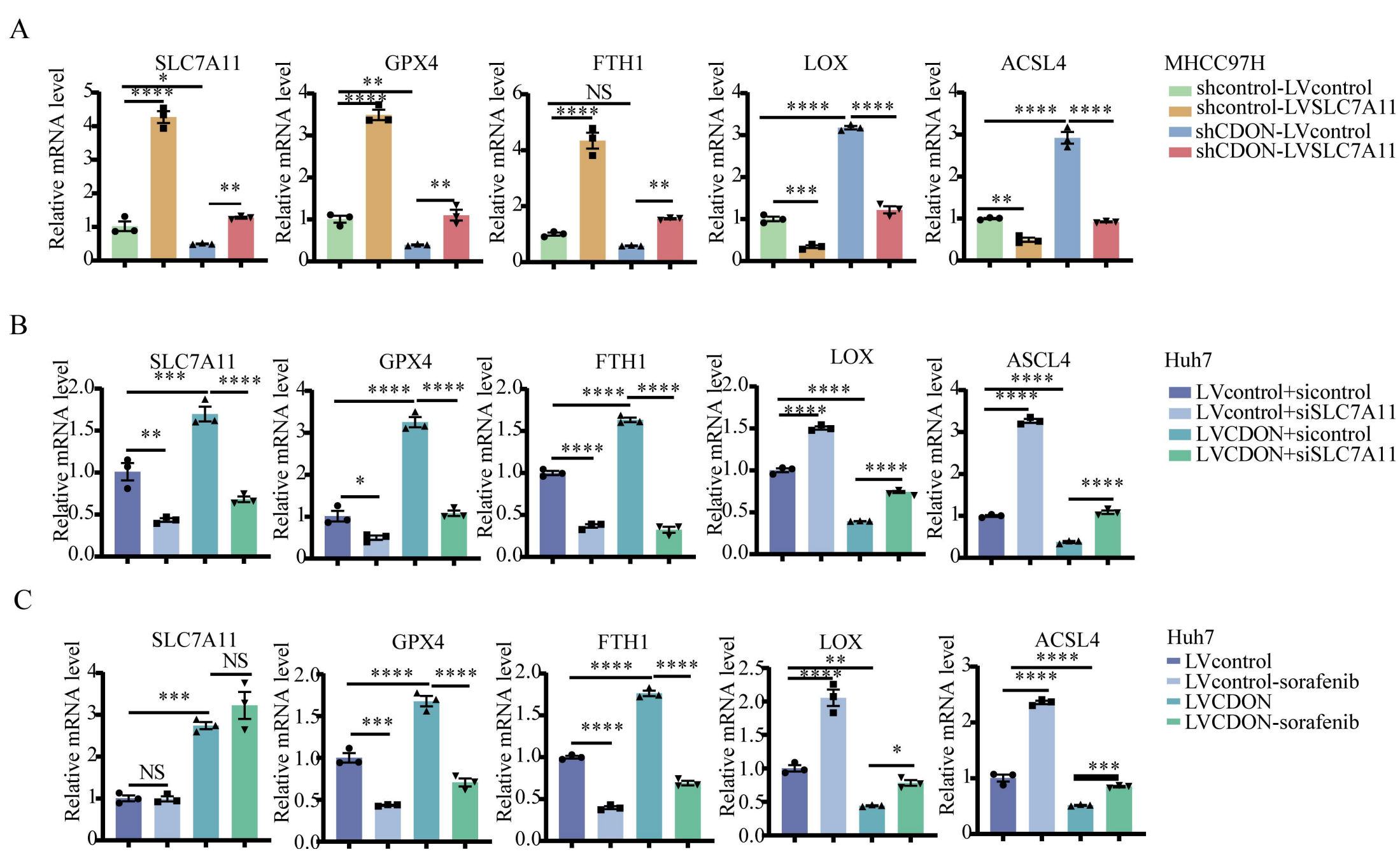

Figure S4 CDON and SLC7A11 or sorafenib jointly regulate ferroptosis in HCC.

A-B. qRT-PCR detection of the expression of ferroptosis-related molecules (SLC7A11, GPX4, FTH1, LOX, ACSL4) in HCC cells under the combined regulation of CDON and SLC7A1 ($n$ = 3).

C. qRT-PCR detection of the expression of ferroptosis-related molecules in HCC cells under the combined regulation of CDON and sorafenib ($n$ = 3).

Figure S5

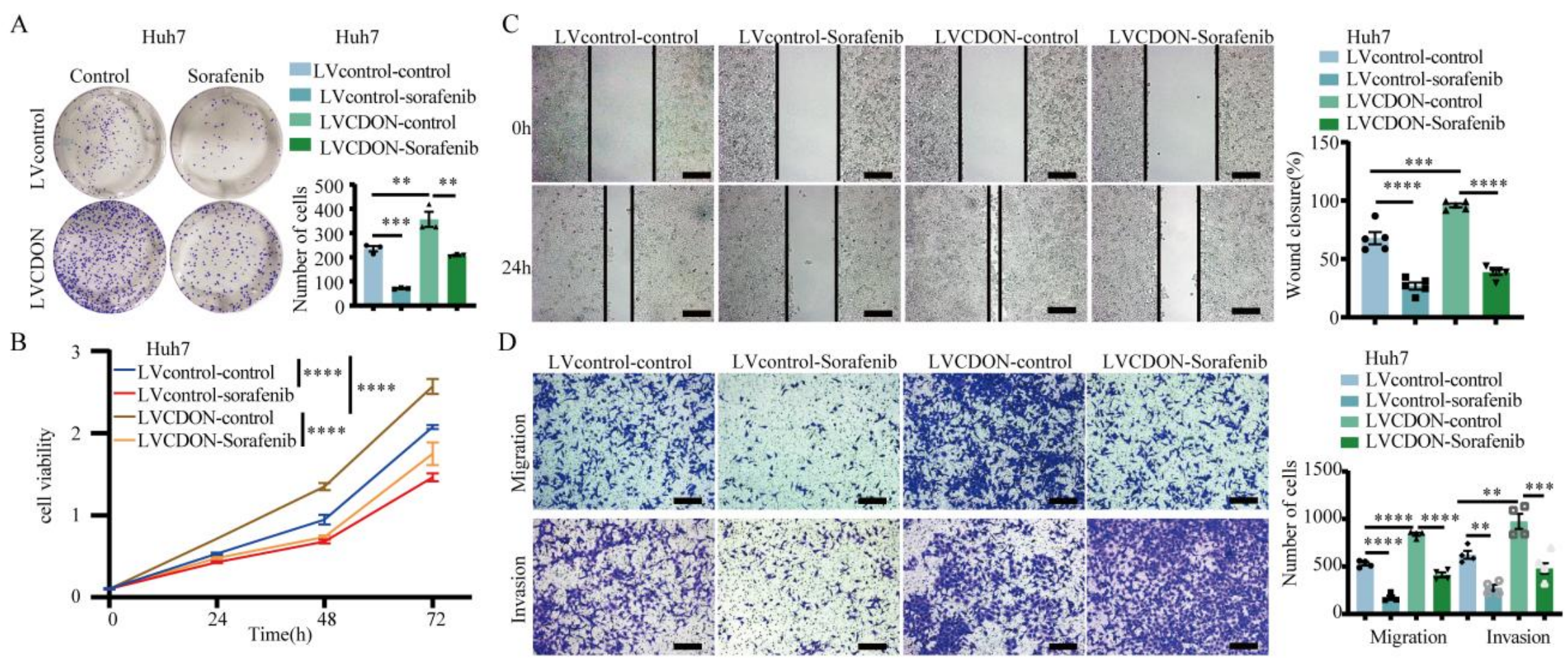


Figure S5 CDON promotes the resistance of HCC to sorafenib

A-B. Colony formation and CCK-8 assays were used to detect the proliferation ability of Huh7-LVCDON cells treated with Sorafenib (5 μM) for 24 hours ($n$ = 3).

C A Wound healing assay was used to detect the migration ability in the indicated cells. Scale bar: 100 μm ($n$ = 5).

D The migratory and invasive capacity of the indicated cells was analyzed by Transwell assay. Scale bar: 100 μm ($n$ = 4).

Figure S6

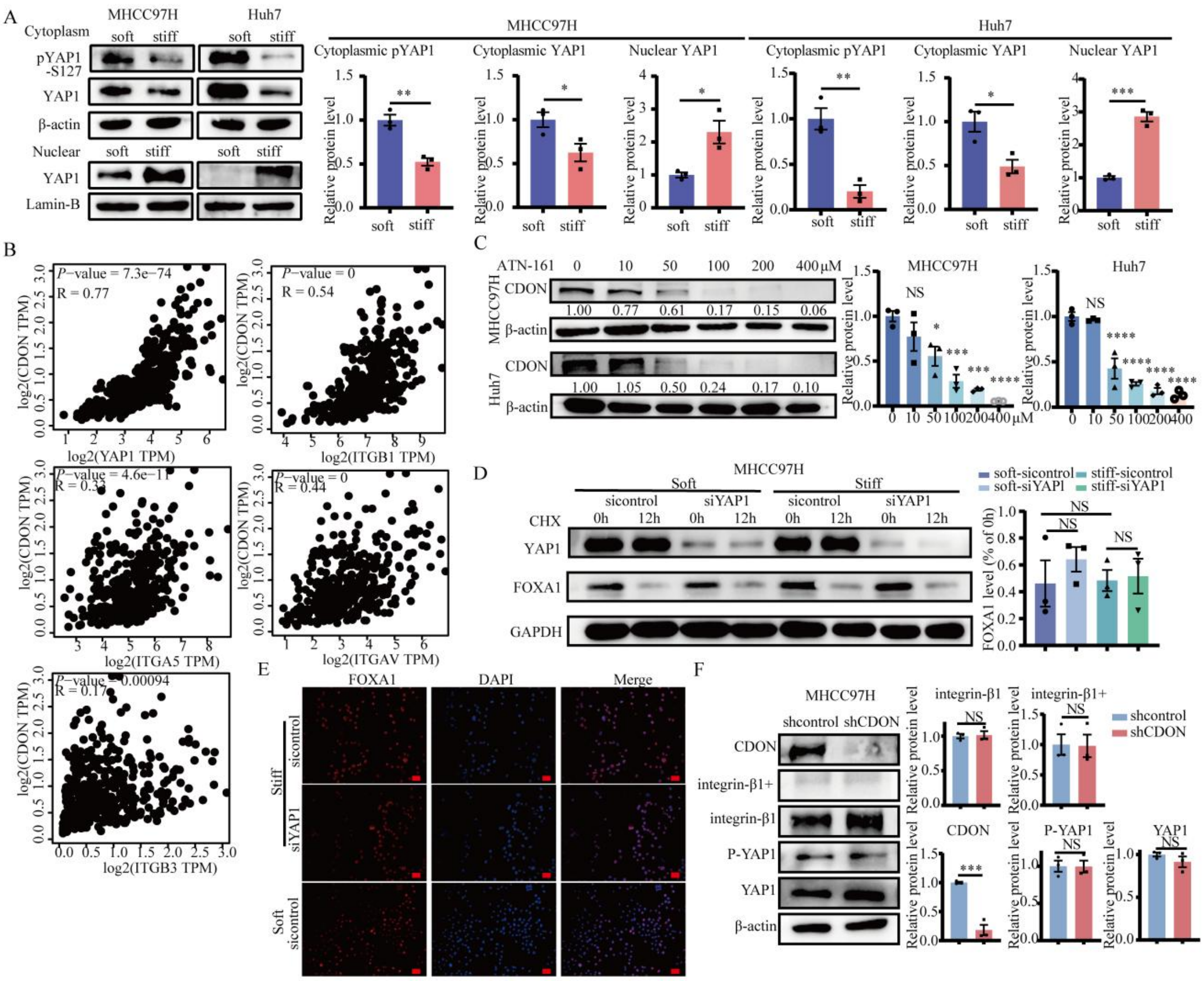


Figure S6 ECM stiffness regulates CDON expression through the integrins/YAP1-FOXA1 axis in HCC cells.

A MHCC97H and Huh7 cells were cultured on soft and stiff ECM conditions. Western blot was used to detect the protein levels of YAP1 and p-YAP1 in the cytoplasm and nucleus of MHCC97H and Huh7 cells ($n$ = 3).

B Correlation analysis diagram of CDON and key molecules involved in the signal transduction of ECM stiffness, including ITGB1 (integrin β1), ITGB3 (integrin β3), ITGA5 (integrin α- 5), ITGAV (integrin α- v), and YAP1 by GEPIA (http://gepia.cancer - pku.cn/).

C Western blot detected the expression of CDON after HCC cells were treated with

ATN-161 (0, 10, 50, 100, 200, 400 μM) (*n* = 3).

D MHCC97H cells were cultured on soft and stiff ECM conditions. Then, they were transfected with siYAP1. Western blot was used to detect the expression of YAP1 and FOXA1 in the treated cells after treatment with protein synthesis inhibitor, CHX (10 μM, 12h) (*n* = 3).

E Immunofluorescence assay for the expression of FOXA1 in the indicated HCC cells. Scale bar: 50 μm.

F Western blot detected the expression of YAP1, p-YAP1, integrins, and their active forms(Integrin β1+) (*n* = 3).

Figure S7

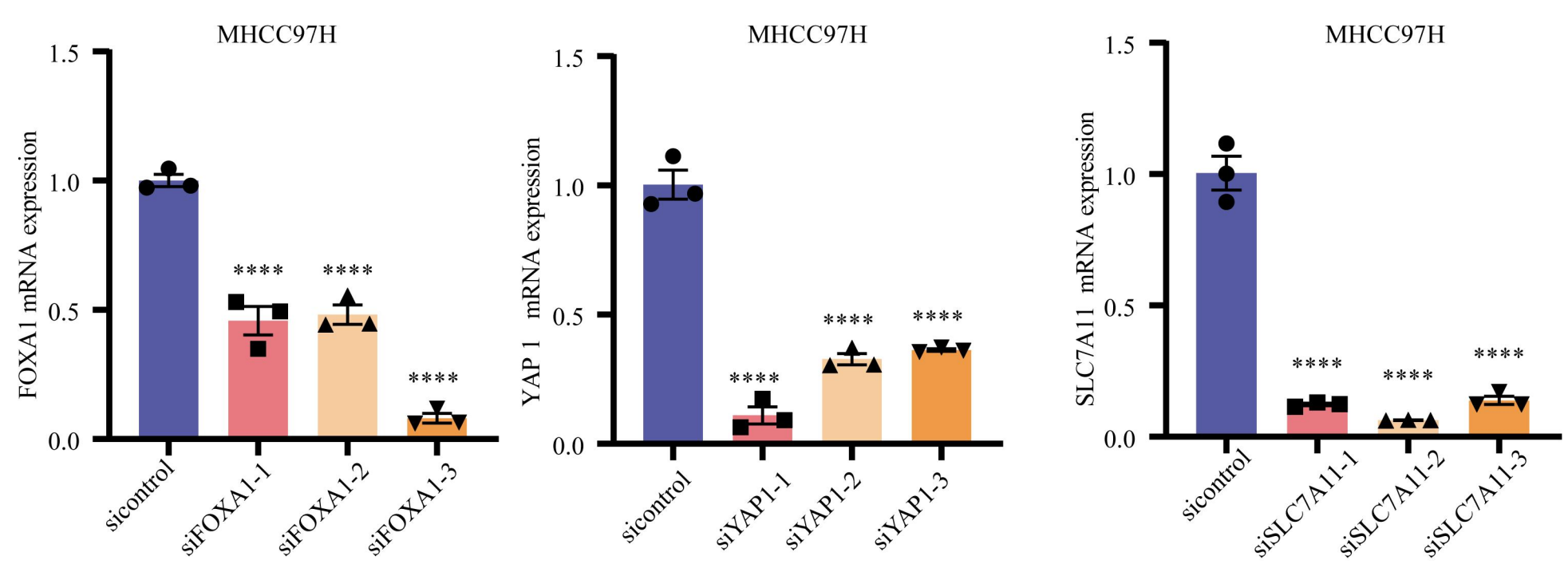


Figure S7 The qRT-PCR detection diagram shows the knockdown efficiency of YAP1, FOXA1, and SLC7A11 (*n* = 3).

Supplementary Tables

Table S1 Cox regression analysis

| Variable | Univariate COX analysis | | Multivariate COX analysis | |
|---|---|---|---|---|
| | HR (95% CI) | P value | HR (95% CI) | P value |
| CDON (High vs. Low) | 3.241 (1.827-6.132) | 0.00003 **** | 3.305 (1.803-6.422) | 0.00007 **** |
| The maximum diameter of the tumor (>5cm vs. ≤5cm) | 1.047 (0.6331-1.755) | 0.860 | 0.9016(0.5287-1.553) | 0.706 |
| Differentiation (Poor vs. Well/Mod) | 1.908 (1.114-3.263) | 0.019* | 2.068 (1.190-3.594) | 0.010* |
| Age (>52.5(median) vs. ≤52.5) | 2.292 (1.359-3.920) | 0.002* | 1.779(1.029-3.112) | 0.039* |
| AFP (>20 vs. ≤ 20 ng/mL) | 1.243 (0.7486-2.054) | 0.397 | 1.769 (1.033-3.026) | 0.038* |

Table S2 Research Resource Identifiers (RRIDs) of the cell line

| Cell line name | Research Resource Identifiers (RRIDs) |
|---|---|
| MHCC97H | CVCL_4972 |
| PLC/PRF/5 | CVCL_0485 |
| SNU-398 | CVCL_0077 |
| Huh 7 | CVCL_0336 |
| THLE2 | CVCL_3803 |
| THLE3 | CVCL_3804 |
| HEK-293T | CVCL_0063 |
| Hepa 1-6 | CVCL_0327 |

Table S3 Knockdown shRNA/siRNA sequence list

| | Sequences (5'->3') | |
|---|---|---|
| The siRNA of the negative control | sense | UUCUCCGAACGUGUCACGUTT |
| | antisense | ACGUGACACGUUCGGAGAATT |
| shcontrol | TTCTCCGAACGTGTCACGT | |
| SiYAP1-1 | GGUCAGAGAUACUUCUUAAAU | |
| SiYAP1-2 | GCUCAGAUCCUUUCCUUAACA | |
| SiYAP1-3 | GGUGAUACUAUCAACCAAAGC | |
| SiFOXA1-1 | CCGGCAACAUGUUCGAGAA | |
| SiFOXA1-2 | CCAGCGACUGGAACAGCUAG | |
| SiFOXA1-3 | CGCUGAGCGAGAUCUACCA | |
| SiSLC7A11-1 | CCUGUCACUAUUUGGAGCUUU | |
| SiSLC7A11-2 | CCUGCGUAUUAUCUCUUUAUU | |
| SiSLC7A11-3 | CCCUGGAGUUAUGCAGCUAAU | |
| ShCDON-1 | GCTTCTGAATATCCTGTCAAA | |
| ShCDON-2 | GCGATACTCAGATCATGCTAA | |
| ShCDON-3 | GCCTTCAAAGTCGAATATAAA | |

Table S4 Expected modulus of elasticity after polymerization of relative acrylamide and bis-acrylamide concentrations.

| Acrylamide % | Bisacrylamide % | 40% acrylamide (mL) | 2% bisacrylamide (mL) | Water (mL) | Young ’ S modulus, E ± SD (kPa) |
|---|---|---|---|---|---|
| 3 | 0.06 | 0.75 | 0.3 | 8.95 | 0.48±0.16 |
| 4 | 0.1 | 1 | 0.5 | 8.5 | 2.01±0.75 |
| 10 | 0.1 | 2.5 | 0.5 | 7 | 10.61 |
| 8 | 0.264 | 2 | 1.32 | 6.68 | 19.66 ±1.19 |
| 8 | 0.48 | 2 | 2.4 | 5.6 | 40.40± 2.39 |

Table S5 List of Antibodies

| Antibody name | Source and dilution ratio |
|---|---|
| anti-CDON | Proteintech,17634-1-AP,1:1000 |
| anti-HMGB1 | Proteintech,10829-1-AP,1:6000 |
| anti-p53 | Proteintech,10442-1-AP,1:10000 |
| anti-SLC7A11 | ABclonal, A13685,1:3000 |
| anti-YAP1 | ABclonal, A21216,1:20000 |
| anti-P-YAP1-S127 | ABclonal, AP0489,1:1000 |
| anti-Lamin B 1 | Proteintech,12987-1-AP,1:20000 |
| anti-FOXA1 | Proteintech,20411-1-AP,1:3000 |
| anti-β-actin | ABclonal, AC026,1:100000 |
| anti-GAPDH | ABclonal, A19056,1:100000 |
| anti-Flag | ABclonal,AE092，1：200(IP), 1:400(IF) |
| anti-GST | ABclonal, AE077,1:4000 |
| anti-LOX | Zenbio, R381849,1:1000 |
| anti-FTH1 | ABclonal, A19544,1:4000 |
| anti-ACSL4 | ABclonal, A20414,1:1000 |
| anti-GPX4 | Proteintech,67763-1-lg,1:1000(IHC),1:2000(WB) |
| anti-CD31 | Abcam, ab182981,1:2000 |
| anti-CD68 | Abcam, ab303565,1:1000 |
| anti-F4/80 | CST,70076T,1:200 |
| anti-ki67 | ABclonal, A20018,1:1000 |
| anti-Histone H3 | ABclonal, AC070, 1:10000 |
| anti- Integrin β1 | Zenbio, R24729, 1:4000 |
| anti-Hust-4 | Sigma-Aldrich，MAB2079Z, 1:500 |
| anti-CDON | GeneTex, GTX105422, 1:200 |
| anti-FOXA1 | CST, #53528, 1:50(ChIP), 1:400(IF) |
| Anti-Ubiquitin | CST, #3936, 1:1000 |

| | |
|---|---|
| anti-Cleaved caspase-3 | HUABIO,ET1602-47,1:1000 |
| anti-LC3B | HUABIO,ET1701-65,1:2000 |
| anti-P62 | Proteintech, 18420-1-AP,1:10000 |
| anti-Phospho-MLKL(S358) | HUABIO,HA724063,1:5000 |
| anti-MLKL | HUABIO,ET1601-25,1:5000 |
| anti-RIPK3 | HUABIO,HA722183,1:2000 |

Table S6 Primer sequence list

| The name of the primer | | sequence (5'->3') |
|---|---|---|
| Primers for real-time PCR | | |
| (HUMAN)GAPDH | forward primer | TCATTTCCTGGTATGACAACGA |
| (HUMAN)GAPDH | reverse primer | GTCTTACTCCTTGGAGGCC |
| (HUMAN)FTH1 | forward primer | CTTCCTTCAGGATATCAAGAAACC |
| (HUMAN)FTH1 | reverse primer | TCCAAATGTAATGCACACTCC |
| (HUMAN)SLC7A11 | forward primer | GGTCCATTACCAGCTTTTGTACG |
| (HUMAN)SLC7A11 | reverse primer | AATGTAGCGTCCAAATGCCAG |
| (HUMAN) GPX4 | forward primer | GTGGATGAAGATCCAACCC |
| (HUMAN) GPX4 | reverse primer | TTGTCGATGAGGAACTTGG |
| (HUMAN) ACSL4 | forward primer | CATCCCTGGAGCAGATACTCT |
| (HUMAN) ACSL4 | reverse primer | TCACTTAGGATTTCCCTGGTCC |
| (HUMAN)LOX | forward primer | CAAGGGACATCAGATTTCTTACC |
| (HUMAN)LOX | reverse primer | CCATACTGTGGTAATGTTGATGAC |
| (HUMAN)P53 | forward primer | GAGGTTGGCTCTGACTGTACC |
| (HUMAN)P53 | reverse primer | TCCGTCCCAGTAGATTACCAC |
| (HUMAN)β-actin | forward primer | CCTTCCTGGGCATGGAGTC |
| (HUMAN)β-actin | reverse primer | TGATCTTCATTGTGCTGGGTG |
| (HUMAN)FOXA1 | forward primer | GCAATACTCGCCTTACGGCT |
| (HUMAN)FOXA1 | reverse primer | TACACACCTTGGTAGTACGCC |
| (HUMAN)YAP1 | forward primer | TGTCCCAGATGAACGTCAC |
| (HUMAN)YAP1 | reverse primer | CAGGAAGAGGACCTGAAGC |
| (HUMAN)CDON | forward primer | GAGTTCAGACTTGGCACCT |
| (HUMAN)CDON | reverse primer | CAATGCAGTACTACAGGTCCA |

Table S7 Primer sequence list

| Primers used for ChIP | | sequence (5'->3') |
|---|---|---|
| distant region | forward primer | TTGGGAGAGGGACAGAGGAG |
| distant region | reverse primer | TGGGCTTCAACAAGTGAGCA |
| binding site 1 | forward primer | GTGTTCACACAGCTCCCTG |
| binding site 1 | reverse primer | TGCAGTCCCAAGCTTCAAAGA |
| binding site 2 | forward primer | TGAAGCTTGGGACTGCACAT |
| binding site2 | reverse primer | TGCCATTTGAATTCACTATGCCT |
| binding site 3 | forward primer | GGCACTGTGAGATCTGGAGG |
| binding site 3 | reverse primer | ACCACACCCAGCCAGAAAAA |
| binding site 4 | forward primer | TGAGCCAAGATCGCACCATT |
| binding site 4 | reverse primer | ATGCCAGTCCTCCCTTACCT |
| binding site 5 | forward primer | TGAGTGGCAGGTGCTTTTCA |
| binding site 5 | reverse primer | AACTCAGGGGCTAGGAGAGG |
| binding site 6 | forward primer | GGTGGTGGGGGAATCAGATG |
| binding site 6 | reverse primer | CCCCTCGCCTCTACCTAAGT |
| human CTGF | forward primer | TTCTGTGAGCTGGAGTGTGC |
| human CTGF | reverse primer | GCCAATGAGCTGAATGGAGT |

Table S8 Online analysis websites used in this study

| website name | Website address |
|---|---|
| Kaplan-Meier Plotter | https://kmplot.com/analysis/ |
| GEPIA | http://gepia.cancer-pku.cn/index.html |
| UALCAN | https://ualcan.path.uab.edu/ |
| UCSC | https://genome.ucsc.edu/ |
| Cistrome DB | http://cistrome.org/db/#/ |
| STRING | https://cn.string-db.org/cgi/input?sessionId=bCgNvE5Eck1I&input_page_show_search=on |
| JASPAR | https://jaspar.elixir.no/ |

## Supplementary Methodology

Colony formation assay and Cell Counting Kit-8 (CCK-8) assay

The cells were treated and then inoculated into 6-well plates ($1 \times 10^3$ cells per well) in a 37°C, 5% $CO_2$ incubator for 2 weeks. Then, the culture medium was removed, and the cells were fixed with 4% polyformaldehyde. After staining with 0.5% crystal violet, the

cells were photographed.

We inoculated the cells with different treatments into 96-well plates ($3 \times 10^3$ cells per well), and used the CCK-8 (BMU106, Abbkine) according to its instructions to measure the absorbance at 450 nm using a microplate reader for the examination.

Transwell assays

Transwell assays were conducted using an 8-μm aperture chamber (Promoter Biotechnology, Wuhan, China) to detect cell migration and invasion. 12% FBS-containing DMEM was added to the bottom chamber, while the top chamber was coated with Matrigel (60 μl, diluted 1:5 with DMEM, ABW) for the invasion assay. $4 \times 10^4$ (migration experiment) and $8 \times 10^4$ (invasion experiment) cells were suspended in serum-free DMEM and added to the top chamber and cultured at 37°C with 5% $CO_2$ for 24 h and 48 h, respectively. The cells were fixed in 4% paraformaldehyde for 20 min, stained with 0.5% crystal violet for 20 min. The cells that failed to penetrate the pores were removed with a cotton swab, and then images were captured using an Olympus microscope (randomly selecting at least 4 areas for imaging).

Immunohistochemical Staining (IHC)

Sections of the paraffin were baked in the oven at 65 °C for 2h and then followed by dewaxing with an environmentally friendly dewaxing solution and a series of ethanol gradients (100–70%). The antigen recovery was performed using sodium citrate buffer, followed by high-temperature microwave treatment for 15 minutes and cooling at room temperature. Next, follow the instructions of the mouse SP detection kit (ZSGB-BIO, Beijing, China, SP-9002) and the DAB immunohistochemical detection kit (Biosci, Wuhan, China) for the operation. The primary antibody used in Table S5. IHC scores were performed by two independent observers who were blinded to the clinical outcome. The percentage of positive cells was evaluated on a scale of 0 to 4: 0 (negative), 1 (1%-25%), 2 (26%-50%), 3 (51%-75%), or 4 (76%-100%). The immunostaining intensity

was scored on a scale of 0 to 3: 0(negative), 1 (weak), 2 (medium), or 3 (strong). The final IHC scores (0~12) were calculated by multiplying the above two scores. The optimal cut-off value for CDON expression was determined by X-tile based on overall survival (OS), which categorized patients into high- and low-expression groups. Each case was ultimately considered“low” if the final score ranged from 0~4, and“high” if the final score ranged from 5~12.

Quantitative Real-Time polymerase chain reaction (qRT-PCR),

Total RNA was extracted from liver cell lines and tissues using TRIzol reagent (Invitrogen). Next, reverse transcription polymerase chain reaction (RT-PCR) was performed using SuperMix to synthesize complementary DNA. qRT-PCR for mRNA expression levels was carried out using SYBR qPCR Master Mix. SYBR qPCR Master Mix and SuperMix were obtained from Vazyme (Nanjing, China). The primers used for real-time PCR can be found in Table S6.

Extraction of cytoplasmic and nuclear proteins

Use the Nuclear and Cytoplasmic Protein Extraction Kit (Beyotime, P0027) to separate the cytoplasmic and nuclear proteins, and then proceed with the subsequent experiments.

Bioinformatic analysis

This study utilized the online websites listed in Table S8.
